# Moderate Intensity Statins Plus Ezetimibe Combination Therapy Versus High Intensity Statins Monotherapy After Percutaneous Coronary Intervention: A Systematic Review and Meta‐Analysis

**DOI:** 10.1002/clc.70369

**Published:** 2026-06-15

**Authors:** Emad Uddin Sajid, Muhammad Hasnain Azeem, Alishba Fatima, Syeda Masooma Jafri, Danish Hassan, Taha Ibrahim, Kashish Zehra Manjee, Syed Rayyan Ahmed, Tooba Ali, Rida Shakeel, Muhammad Usman, Raghabendra Kumar Mahato, Danish Bawa

**Affiliations:** ^1^ Department of Medicine Dow University of Health Sciences Karachi Pakistan; ^2^ Gandaki Medical College Teaching Hospital and Research Center Pokhara Nepal; ^3^ Midwest Heart and Vascular Specialist HCA Midwest Overland Park Kansas USA

**Keywords:** acute coronary syndrome, ezetimibe, MACE, myocardial infarction, PCI, statins

## Abstract

**Background:**

High‐intensity statins are standard therapy after percutaneous coronary intervention (PCI) for acute coronary syndrome (ACS), yet tolerability concerns and residual cardiovascular risk remain important challenges. Moderate‐intensity statin therapy combined with ezetimibe has emerged as a potential alternative, although its comparative effectiveness after PCI remains uncertain.

**Methods:**

We performed a systematic review and meta‐analysis according to PRISMA guidelines. PubMed, Cochrane Library, and ClinicalTrials.gov were searched through September 2025 for studies enrolling adults undergoing PCI and comparing moderate‐intensity statin plus ezetimibe therapy with high‐intensity statin therapy. The primary outcome was major adverse cardiovascular events (MACE), defined as all‐cause mortality, myocardial infarction (MI), stroke, and revascularization. Secondary outcomes included individual cardiovascular endpoints and safety outcomes such as new‐onset diabetes and rhabdomyolysis. Random‐effects models were used to calculate pooled risk ratios (RRs).

**Results:**

Nine studies involving 179 621 patients were included, the majority being observational studies. No significant difference was observed in MACE between treatment strategies (RR 0.96, 95% CI 0.81–1.12). Modest reductions were observed in cardiovascular mortality (RR 0.83, 95% CI 0.71–0.98) and MI (RR 0.74, 95% CI 0.58–0.95), although findings were non‐robust with wide prediction intervals. Revascularization rates were similar between groups. Subgroup analyses suggested lower heterogeneity in intermediate follow‐up and non‐ACS populations.

**Conclusions:**

Moderate‐intensity statin plus ezetimibe therapy may provide cardiovascular outcomes comparable to high‐intensity statins after PCI. However, persistent heterogeneity limits certainty, and large randomized trials are needed to confirm long‐term efficacy and safety.

## Graphical abstract

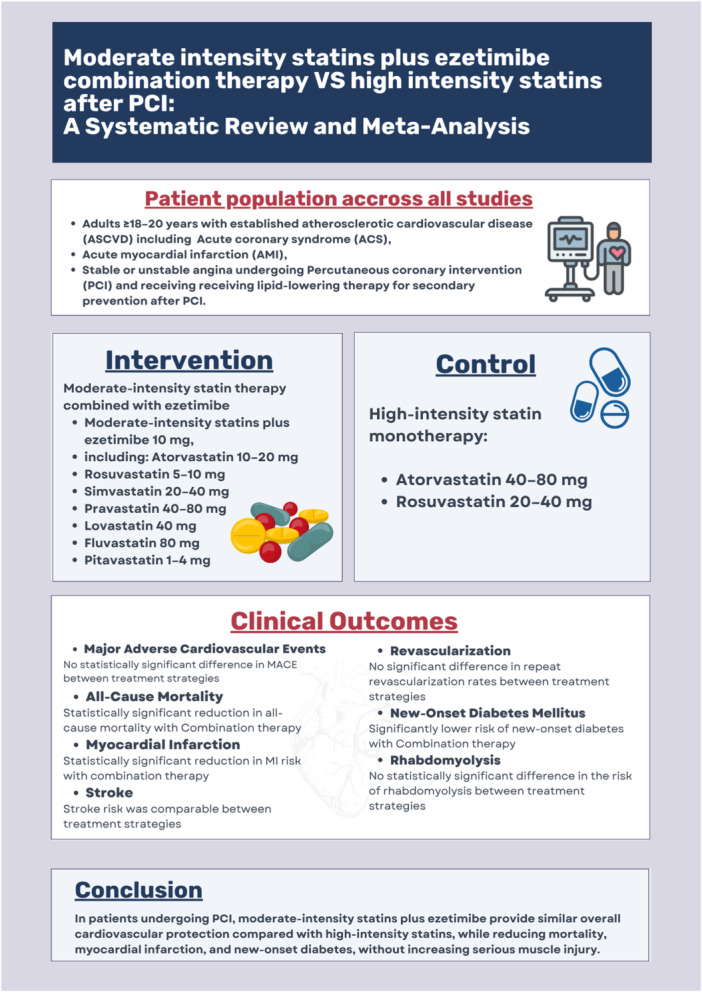

## 1 Introduction

Acute coronary syndrome (ACS), including ST‐elevation myocardial infarction (STEMI), non‐ST‐elevation myocardial infarction (NSTEMI), and unstable angina, continues to be among the major causes of mortality and morbidity worldwide. Ischemic heart disease, which ACS forms a significant part of, resulted in an estimated nine million deaths and almost 188 million disability‐adjusted life years in 2021 [1]. Percutaneous coronary intervention (PCI) has become the primary therapeutic strategy for ACS, and most significantly in STEMI, where early reperfusion is the key to minimizing death and risk of recurrent ischemic complications [2].

Increased low‐density lipoprotein cholesterol (LDL‐C) is one of the leading precipitating causes of ACS and atherosclerotic cardiovascular disease (ASCVD). A comprehensive study, encompassing over 90 000 individuals, has shown that each 1.0 mmol/L rise in LDL‐C was associated with a 34% heightened risk of myocardial infarction (MI) and a 16% increased risk of ASCVD, with the association being particularly pronounced in individuals aged 70–100 years [3]. Conversely, data from a large post‐ACS cohort study has shown that a 1 mmol/L reduction in LDL‐C 1‐year post‐ACS was linked to a 23% reduction in the risk of major adverse cardiovascular events (MACE) [4].

Patients who undergo PCI for ASCVD are classified as a very high‐risk group for recurrent adverse cardiovascular events. Lipid‐lowering therapy is the standard approach for secondary prevention of cardiovascular disease in these patients. 2019 European guidelines recommend both ≥ 50% relative reduction from baseline in LDL‐C and an LDL‐C level < 1.4 mmol/L (55 mg/dL) in very high‐risk patients. To achieve that goal, high‐intensity statin therapy is recommended as first‐line treatment [5, 6]. However, there are discrepancies between guideline recommendations and implementation in real‐world practice [7, 8]. This may be attributed to potential side effects or intolerance of patients to high‐intensity statins [9, 10]. Furthermore, studies have demonstrated that many patients who undergo PCI do not reach the target LDL‐C levels [11]. Increasing the statin dose to address this issue is associated with adverse effects such as a dose‐dependent increase in the risk of new‐onset diabetes over the long term [12, 13]. Moreover, doubling the statin dose yields only an additional 6%–7% reduction in LDL‐C [14].

These limits have sparked growing interest in combination therapies. An early or initial combination of ezetimibe with a lower intensity statin, as opposed to maximizing statin potency until the onset of intolerance, may offer equal or greater LDL‐C reduction with fewer side effects, particularly myopathy, and increased compliance [15]. Clinical trials, such as the IMPROVE‐IT trial, confirmed that the addition of ezetimibe to statin therapy lowered LDL‐C levels and improved cardiovascular events in ACS patients [16]. More recently, the RACING trial demonstrated that the addition of ezetimibe to medium‐intensity statins was noninferior to high‐intensity statin treatment in preventing MACE in ASCVD patients. Furthermore, combination lipid‐lowering therapy was correlated with increased drug adherence and greater LDL‐C reduction than high‐intensity statin monotherapy [17].

Against this background, this meta‐analysis seeks to provide a comprehensive comparison of these two prominent lipid‐lowering strategies—high‐intensity statins versus moderate‐intensity statins plus ezetimibe—in patients undergoing PCI for ACS. The goal is to evaluate and contrast the impact of these treatment regimens on critical clinical outcomes, including all‐cause mortality, MI, stroke, revascularization procedures, and cardiovascular death.

The goal of this meta‐analysis is to reveal how these lipid‐lowering strategies influence long‐term survival and reduce the risk of recurrent cardiovascular events in post‐PCI patients. Our findings will provide invaluable insights into the most effective approach for optimizing lipid control after PCI, offering clinicians clear evidence on the optimal use of statin therapy, potentially combined with ezetimibe, to enhance patient outcomes. This comparison could reshape the post‐PCI treatment paradigm, helping to refine strategies that improve both survival rates adherence and quality of life.

## 2 Methods

We followed the Preferred Reporting Items for Systematic Reviews and Meta‐Analyses (PRISMA) guidelines to perform the study [18]. The study was registered on PROSPERO and is available via [CRD420251248305].

### 2.1 Search Strategy and Study Selection

To identify relevant studies, we searched PubMed AND Cochrane databases from inception to September 2025. No language restrictions were applied. We also searched ClinicalTrials.gov and screened reference lists of eligible studies and other related studies to identify further publications pertinent to our analysis. The search terms included “moderate intensity statins plus ezetimibe,” “high intensity statins,” “percutaneous coronary intervention,” “PCI,” as well as specific drug combinations such as “Atorvastatin plus Ezetimibe,” “Rosuvastatin plus Ezetimibe,” “Simvastatin plus Ezetimibe,” “Pravastatin plus Ezetimibe,” “Lovastatin plus Ezetimibe,” and “Fluvastatin plus Ezetimibe.” Additional terms covered both moderate‐ and high‐intensity statin formulations, including “atorvastatin,” “rosuvastatin,” “pravastatin,” “lovastatin,” and “fluvastatin,” combined with procedural descriptors such as “percutaneous coronary angioplasty,” “coronary stenting,” “primary PCI,” “PTCA,” and “endovascular revascularization.” Detailed search terms are provided in the Supporting Information S1: Table 1.

Two investigators independently performed the database searches. All records retrieved from the searches were imported into EndNote for deduplication. All the screening was also conducted in EndNote. Initial title and abstract screening and subsequent full‐text screening of potentially relevant abstracts were conducted independently by two reviewers, and any discrepancies were resolved by consensus with a third investigator. The full texts of eligible studies were then thoroughly examined for further quality assessment.

### 2.2 Eligibility Criteria

For this meta‐analysis, we included studies that enrolled adult patients undergoing PCI. Eligible interventions consisted of moderate‐intensity statins administered in combination with ezetimibe. The comparator group comprised patients treated with high‐intensity statins alone. We considered randomized controlled trials (RCTs), cohort studies, and other observational designs that reported clinically relevant outcomes after PCI, such as cardiovascular events, mortality, restenosis, or lipid profile changes. Studies were excluded if they were case reports, editorials, reviews, animal studies, or conference abstracts lacking sufficient data, if they did not provide a clear high‐intensity statin comparator group, or if the intervention did not involve ezetimibe in combination with statins. Eligibility criteria based on the Population, Intervention, Comparator, and Outcomes (PICO) framework are summarized in Supporting Information S1: Table 3.

After selecting full‐text articles based on quality, we conducted detailed assessments and data syntheses.

### 2.3 Data Extraction

All the relevant data were extracted from the included studies by two investigators. We extracted relevant data including author, publication year, study design, and sample sizes. Baseline characteristics were collected, including demographics (age, sex), cardiovascular risk factors (hypertension, diabetes, smoking status, dyslipidemia, obesity), and clinical comorbidities such as prior MI, acute MI, unstable or stable angina, heart failure, prior PCI, prior coronary artery bypass grafting (CABG), previous ischemic stroke, prior intracranial hemorrhage, chronic kidney disease, chronic obstructive pulmonary disease (COPD), peripheral artery disease, atrial fibrillation, malignancy, chronic liver disease, peptic ulcer disease, and rhabdomyolysis. We additionally extracted medication at baseline (aspirin, P2Y12 inhibitors, ACE inhibitors/ARBs, β‐blockers, calcium channel blockers, statins), number of implanted stents, duration of dual antiplatelet therapy, lipid parameters (LDL‐C), and Charlson comorbidity index. Baseline characteristics are summarized in Table 1, while baseline and achieved LDL‐C levels across the included studies are summarized in Table 2.

**Table 1:** Baseline characteristics of studies.

Author (year) Study characteristics	Seo Young Sohn [21]	Seung‐Jun Lee (2024)	Seung‐Jun Lee [23]	Juwon Kim (2021)	Kihyun Kim [25]	Mi Seon Ji [26]	Ji‐Yong Jang [27]	Eun Ho Choo [28]	JongII Park [29]
Study design	Retrospective cohort	Retrospective cohort	Retrospective cohort	Cohort study	Cohort study	Propensity matching analysis	Cohort study	Cohort study	Post hoc analysis (RACING trial)
Intervention group (*n*)	4682	6164	10 794	922	616	1249	10 723	7161	1258
Control group (*n*)	2102	25 802	61 256	19 148	9292	2271	10 723	7161	1239
Follow‐up duration	Int: 4.2 ± 1.1 years Con: 4.0 ± 1.2 years	Int/Con: 2.9 ± 0.3 years	Int/Con: 2.9 ± 0.4 years	3, 6, 12 months	Int: 1.54 ± 0.54 years Con: 2.5 ± 1.1 years	12 months	Int: 1028 ± 654 days Con: 1026 ± 645 days	Int/Con: 2.7 years	2 months, 6 months, then yearly
Demographics									
Male sex—*n* (%)	Int: 3071 (65.6) Con: 1539 (73.2)	Int: 4455 (72.3) Con: 19 573 (75.9)	Int: 7867 (72.9) Con: 44 999 (73.5)	Int: 604 (65.5) Con: 14 254 (74.4)	Int: 509 (82.6) Con: 7801 (83.9)	Int: 945 (76.0) Con: 1778 (78.5)	Int: 7544 (70.4) Con: 7590 (70.8)	Int: 5131 (71.7) Con: 5180 (72.3)	Int: 994 (79.0) Con: 971 (78.4)
Age (years) mean ± SD	Int: 65.8 ± 9.9 Con: 63.4 ± 9.6	Int: 61.9 ± 10.9 Con: 62.9 ± 11.3	Int: 62.3 ± 10.9 Con: 62.8 ± 11.5	Int: 65 (IQR 57–73) Con: 63 (IQR 55–73)	Int: 59.22 ± 10.88 Con: 59.49 ± 11.07	Int: 61.2 ± 13.19 Con: 61.3 ± 12.90	Intervention: < 40 years: 1015 (9.5) 50 s: 2489 (23.2) 60 s: 3307 (30.8) 70 s: 2714 (25.3) > 80 s: 1198 (11.2) Control: < 40 years: 994 (9.3) 50 s: 2459 (22.9) 60 s: 3311 (30.9) 70 s: 2734 (25.5) > 80 s: 1225 (11.4)	Int: 64.11 ± 10.59 Con: 64.17 ± 10.53	Int: 63.8 ± 9.5 Con: 64.6 ± 9.6
Cardiovascular risk factors									
Diabetes mellitus—*n* (%)	Int: 2775 (59.3) Con: 1228 (58.4)	Int: 3033 (49.2) Con: 11 273 (43.7)	Int: 5007 (46.4) Con: 27 215 (44.4)	Int: 487 (52.8) Con: 8622 (45.0)	Int: 188 (30.5) Con: 2820 (30.3)	Int: 357 (28.6) Con: 690 (30.4)	Int: 3,546 (33.1) Con: 3516 (32.8)	Int: 2374 (33.2) Con: 2396 (33.5)	Int: 524 (41.7) Con: 509 (41.1)
Hypertension—*n* (%)	Int: 3151 (67.3) Con: 1290 (61.4)	Int: 4730 (76.7) Con: 18 509 (71.7)	Int: 8242 (76.4) Con: 46 246 (75.5)	Int: 681 (73.9) Con: 12 859 (67.2)	Int: 546 (88.6) Con: 8351 (89.8)	Int: 589 (47.7) Con: 1036 (46.3)	Int: 9245 (86.2) Con: 9305 (86.8)	Int: 5461 (76.3) Con: 5399 (75.4)	Int: 868 (69.0) Con: 870 (70.2)
Smoking—*n* (%) (current)	Int: 422 (9.0) Con: 213 (10.1)	NR	NR	NR	Int current: 296 (48.1) Con current: 4195 (45.1)	Int: 836 (67.4) Con: 1488 (66.1)	NR	NR	Int: 211 (16.8) Con: 194 (15.7)
Cardiac history									
STEMI—*n* (%)	NR	NR	NR	NR	Int: 116 (18.8) Con: 1909 (20.5)	Int: 799 (64.0) Con: 1347 (59.3)	Int: 1828 (17.0) Con: 1851 (17.3)	NR	NR
NSTEMI—*n* (%)	NR	NR	NR	NR	Int: 220 (35.7) Con: 2820 (30.3)	Int: 450 (36.0) Con: 924 (40.7)	Int: 3076 (28.7) Con: 3089 (28.8)	NR	NR
Stable angina—*n* (%)	Int: 1405 (30.0) Con: 808 (38.4)	NR	NR	Int: 296 (32.1) Con: 4586 (24.0)	NR	NR	NR	Int: 5520 (77.1) Con: 5517 (77.0)	NR
Unstable angina—*n* (%)	Int: 3277 (70.0) Con: 1294 (61.6)	NR	NR	Int: 388 (42.1) Con: 4994 (26.1)	NR	NR	Int: 4563 (42.6) Con: 4538 (42.3)	NR	NR
Heart failure—*n* (%)	NR	NR	NR	Int: 171 (18.5) Con: 3400 (17.8)	NR	NR	Int: 1788 (16.7) Con: 1737 (16.2)	Int: 1004 (14.0) Con: 989 (13.8)	NR
Prior MI—*n* (%)	NR	Int: 2588 (42.0) Con: 10 872 (42.1)	Int: 2360 (21.9) Con: 13 500 (22.0)	Int: 85 (9.2) Con: 2165 (11.3)	Int: 280 (45.4) Con: 4563 (49.1)	NR	Int: 3967 (37.0) Con: 3962 (37.0)	Int: 602 (8.4) Con: 611 (8.5)	Int: 623 (49.5) Con: 607 (49.0)
Prior PCI—*n* (%)	NR	NR	Int: 2005−2009: 2413 (22.4) 2010−2014: 3380 (31.3) 2015−2016: 5002 (46.3) Control: 2005−2009: 11 327 (18.5) 2010−2014: 20 173 (32.9) 2015−2016: 29 756 (48.6)	Int: 904 (98.0) Con: 18 687 (97.6)	NR	NR	Int: 507 (4.7) Con: 459 (4.3)	Int: 695 (9.7) Con: 697 (9.7)	NR
Prior CABG—*n* (%)	NR	Int: 103 (1.7) Con: 336 (1.3)	Int: 119 (1.1) Con: 632 (1.0)	Int: 18 (2.0) Con: 461 (2.4)	Int: 5 (0.8) Con: 42 (0.5)	NR	Int: 521 (4.9) Con: 462 (4.3)	Int: 10 (0.1) Con: 8 (0.1)	Int: 76 (6.0) Con: 64 (5.2)
Comorbidities									
Chronic kidney disease—*n* (%)	Int: 70 (1.5) Con: 30 (1.43)	Int: 401 (6.5) Con: 1427 (5.5)	Int: 523 (4.9) Con: 2877 (4.7)	Int: 47 (5.1) Con: 696 (3.6)	Int: 3 (0.5) Con: 35 (0.4)	Int: 358 (32.7) Con: 530 (26.9)	Int: 270 (2.5) Con: 167 (1.6)	Int: 1120 (15.6) Con: 1099 (15.3)	Int: 134 (10.7) Con: 139 (11.2)
COPD—*n* (%)	NR	NR	NR	NR	NR	NR	NR	Int: 199 (2.8) Con: 195 (2.7)	NR
PAD—*n* (%)	NR	Int: 399 (6.5) Con: 1349 (5.2)	Int: 625 (5.8) Con: 3332 (5.4)	Int: 307 (33.3) Con: 4854 (25.3)	NR	NR	Int: 891 (8.3) Con: 829 (7.7)	Int: 455 (6.4) Con: 466 (6.5)	Int: 30 (2.4) Con: 28 (2.3)
Atrial fibrillation—*n* (%)	NR	NR	NR	NR	NR	NR	NR	Int: 317 (4.4) Con: 297 (4.1)	NR
Prior ischemic stroke—*n* (%)	NR	Int: 969 (15.7) Con: 4488 (17.4)	Int: 1747 (16.2) Con: 9552 (15.6)	Int: 81 (8.8) Con: 2075 (10.8)	NR	NR	NR	Int: 743 (10.4) Con: 733 (10.2)	Int: 73 (5.8) Con: 85 (6.9)
Charlson comorbidity index—mean ± SD	Int: 2.9 ± 2.3 Con: 2.7 ± 2.1	Int: 3.5 ± 2.3 Con: 3.3 ± 2.3	Int: 3.4 ± 2.3 Con: 3.3 ± 2.3	NR	Int: 2.8 ± 1.9 Con: 3.2 ± 2.0	NR	NR	Int: 2.43 ± 1.89 Con: 2.41 ± 1.86	NR
Medications at baseline									
Statin therapy—*n* (%)	NR	Int: High 1285 (20.8) Low–mod 3400 (55.2) None 1479 (24.0) Con: High 11,098 (43.0) Low–mod 5208 (20.2) None 9496 (36.8)	Int: High 2193 (20.3) Low–mod 4892 (45.3) None 3709 (34.4) Con: High 12 246 (20.0) Low–mod 26 396 (43.1) None 22 614 (36.9)	Int: High 5 (0.5) Mod 87 (9.4) Ezetimibe 64 (6.9) Con: High 535 (2.8) Mod 1801 (9.4) Low 29 (0.2) Ezetimibe 136 (0.7)	Int: 616 (100) Con: 9284 (99.9)	NR	NR	Int: High 5102 (71.2) Con: High 5031 (70.3)	Int: High 554 (44.0) High+Ezet 67 (5.3) Mod 441 (35.1) Con: High 548 (44.2) Con: High+Ezet 51 (4.1) Con: Mod 481 (38.8)
Aspirin—*n* (%)	NR	Int: 4675 (75.8) Con: 15 932 (61.8)	Int: 7049 (65.3) Con: 37 845 (61.8)	Int: 874 (94.8) Con: 18 898 (98.7)	NR	NR	NR	Int: 6857 (95.8) Con: 6896 (96.3)	NR
P2Y12 Inhibitor—*n* (%)	NR	Int: 3808 (61.8) Con: 12 005 (46.5)	Int: 5247 (48.6) Con: 28 565 (46.6)	Int: 809 (87.7) Con: 18 330 (95.7)	NR	NR	Int: 1255 (11.5) Con: 1245 (11.6)	Int: Clop 5630 (78.6) Pras 448 (6.3) Tica 779 (10.9) Con: Clop 5638 (78.7) Pras 470 (6.6) Tica 788 (11.0)	NR
ACEI/ARB—*n* (%)	Int: 2070 (44.2) Con: 877 (41.7)	Int: 2966 (48.1) Con: 10 544 (40.9)	Int: 4223 (39.1) Con: 23 728 (38.7)	Int: 470 (51.0) Con: 13 759 (71.9)	Int: ACEI 241 (39.1) ARB 302 (49.0) Con: ACEI 4052 (43.6) ARB 4435 (47.7)	Int: 1056 (87.3) Con: 1951 (95.0)	NR	Int: 3333 (46.5) Con: 3339 (46.6)	NR
Beta‐blocker—*n* (%)	Int: 705 (15.1) Con: 336 (16.0)	Int: 3222 (52.3) Con: 11 384 (44.1)	Int: 4579 (42.4) Con: 25 251 (41.2)	Int: 572 (62.0) Con: 14 837 (77.5)	Int: 505 (81.9) Con: 8195 (88.1)	Int: 1115 (92.0) Con: 2060 (97.6)	NR	Int: 4379 (61.2) Con: 4387 (61.3)	NR
LDL‐C (mg/dL) mean ± SD	NR	NR	NR	NR	Int: 135.9 ± 42.0 Con: 132.8 ± 41.4	Int: 125.2 ± 38.56 Con: 121.9 ± 39.80	NR	NR	Int: 77 (IQR 61–93) Con: 76 (IQR 61–92)

*Note:* Data are presented as *n* (%) for categorical variables and mean ± SD or median (IQR) for continuous variables. Intervention and control group values are listed separately where distinct values were reported in the source publication.

Abbreviations: ACEI, angiotensin‐converting enzyme inhibitor; AF, atrial fibrillation; ARB, angiotensin receptor blocker; BB, beta‐blocker; CABG, coronary artery bypass grafting; CCI, Charlson comorbidity index; CKD, chronic kidney disease; Clop, clopidogrel; COPD, chronic obstructive pulmonary disease; Con, control group; DM, diabetes mellitus; Ezet, ezetimibe; HF, heart failure; HTN, hypertension; Int, intervention group; IQR, interquartile range; LDL‐C, low‐density lipoprotein cholesterol; MI, myocardial infarction; Mod, moderate‐intensity; NR, not reported; NSTEMI, non‐ST‐elevation myocardial infarction; PAD, peripheral artery disease; PCI, percutaneous coronary intervention; Pras, prasugrel; P2Y12, P2Y12 receptor inhibitor; RCT, randomized controlled trial; SA, stable angina; SD, standard deviation; STEMI, ST‐elevation myocardial infarction; Tica, ticagrelor; UA, unstable angina.

**Table 2:** Baseline and achieved LDL‐C levels across included studies.

Study	Treatment group	Baseline LDL‐C (mg/dL)	LDL‐C at follow‐up (mg/dL)	LDL‐C reduction (%)
		Mean ± SD	Mean ± SD	
*Mi Seon Jee* et al. [26]				
Overall population	High‐intensity statin	121.9 ± 39.80	70.8 ± 29.28	41.9
	Simvastatin–EZ	125.2 ± 38.56	73.1 ± 29.76	41.6
Propensity‐score matched	High‐intensity statin	127.2 ± 38.90	72.8 ± 26.15	42.8
	Simvastatin–EZ	126.4 ± 40.04	76.2 ± 23.40	39.7
*Park* et al., *2025 (Prior PCI subgroup, N* = *2497)*				
1‐Year	Moderate‐intensity statin + ezetimibe	77 ± 23.72	57.66 ± 17.79	25.11
	High‐intensity statin monotherapy	76.33 ± 22.98	66.33 ± 18.53	15.07
*Park* et al., *2025 (No prior PCI subgroup, N* = *1283)*				
1‐Year	Moderate‐intensity statin + ezetimibe	92.33 ± 32.61	59.66 ± 19.274	35.38
	High‐intensity statin monotherapy	93.33 ± 34.09	69 ± 20.756	26.06

Abbreviations: ACS, acute coronary syndrome; EZ, ezetimibe; LDL‐C, low‐density lipoprotein cholesterol; PCI, percutaneous coronary intervention; SD, standard deviation.

Outcome data were extracted, including primary outcomes of MACE. Secondary outcomes included all‐cause mortality, cardiovascular death, MI, stroke, and revascularization. Safety endpoints included rhabdomyolysis and new‐onset diabetes mellitus. New‐onset DM was analyzed for patients who have received new anti‐diabetic medications.

### 2.4 Risk of Bias (RoB) Assessment

RoB was evaluated independently by two investigators, with any disagreements resolved by consensus with a third investigator. RCTs were assessed using the Cochrane Risk of Bias tool for randomized trials, version 2 (RoB 2), while non‐randomized studies were evaluated using the ROBINS‐I tool. The RoB 2 framework considered potential bias in the randomization process, deviations from intended interventions, missing outcome data, outcome measurement, and selective reporting [19]. ROBINS‐I assessed bias across seven domains including confounding, selection of participants, classification of interventions, deviations from intended interventions, missing data, measurement of outcomes, and selective reporting [20]. Each study was assigned an overall judgment, and the results were summarized using traffic‐light plots and bar graphs to provide both domain‐specific and overall risk assessments.

### 2.5 Data Synthesis and Statistical Analysis

The meta‐analysis was carried out using RStudio. Data were synthesized using the risk ratio (RR) for dichotomous outcomes. All results were reported with 95% confidence intervals (CIs), and a *p* value of ≤ 0.05 was considered statistically significant. A random‐effects model was used to account for residual clinical and methodological inter‐study heterogeneity. The *I* ^2^ statistic that quantifies statistical heterogeneity was calculated; any value above 50% was taken as a determination of significant heterogeneity. Publication bias was not formally assessed with funnel plots or Egger's regression test, as fewer than 10 studies contributed to most outcomes, which limits the reliability of such methods. All statistical analyses were conducted in RStudio (R version 4.5.1) using the Meta and metabin packages. A two‐sided *p* < 0.05 was considered statistically significant for effect estimates, while *p* < 0.10 was applied for heterogeneity tests.

## 3 Results

A systematic review and meta‐analysis were conducted to compare the efficacy and safety of combination therapy with moderate‐intensity statins plus ezetimibe versus high‐intensity statin monotherapy in patients who underwent PCI. A total of nine studies were included for quantitative synthesis. The primary outcomes assessed were MACE and their individual components.

### 3.1 Study Selection and Characteristics

**Figure 1:**
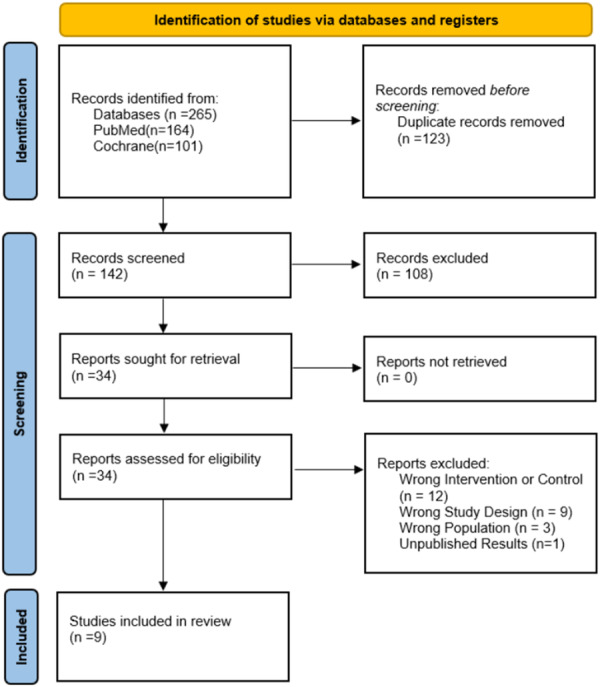
PRISMA flow diagram.

A systematic review identified 265 records, of which nine studies met inclusion criteria for quantitative synthesis (PRISMA flow diagram, Figure 1) [21, 22, 23, 24, 25, 26, 27, 28, 29]. Of these, eight were observational cohort studies, and one was a post hoc analysis of a RCT [29]. One additional study (Pyung Chun Oh 2021) assessed percent atheroma volume and did not report clinical outcomes; it was therefore excluded from pooled analyses but is described narratively.

A total of 179 621 patients were included across the nine studies. Follow‐up duration ranged from 1 to 4 years, with seven studies reporting outcomes at ≥ 2.5 years. RoB was assessed using ROBINS‐I for observational studies and RoB‐2 for the RCT. Four observational studies were rated as having serious RoB (Seo Young Sohn 2025, Seung‐Jun Lee 2024, Mi Seon Ji [26], Kihyun Kim [25]), four as moderate (Seung‐Jun Lee [23], Juwon Kim 2021, Ji‐Yong Jang [27], Eun Ho Choo [28]), and the single RCT (Jong‐II Park [29]) as having moderate RoB (some concerns due to post‐hoc design). No study was rated as low RoB. Detailed study characteristics, including study design, follow‐up duration, intervention regimens, and comparator therapies, are summarized in Supporting Information S1: Table 2.

Baseline characteristics, including LDL‐cholesterol levels, statin regimens, and comorbidity profiles, are summarized in Table 1. Baseline and achieved LDL‐C levels across the included studies are summarized in Table 2.

### 3.2 Quality Assessment

**Figure 2:**
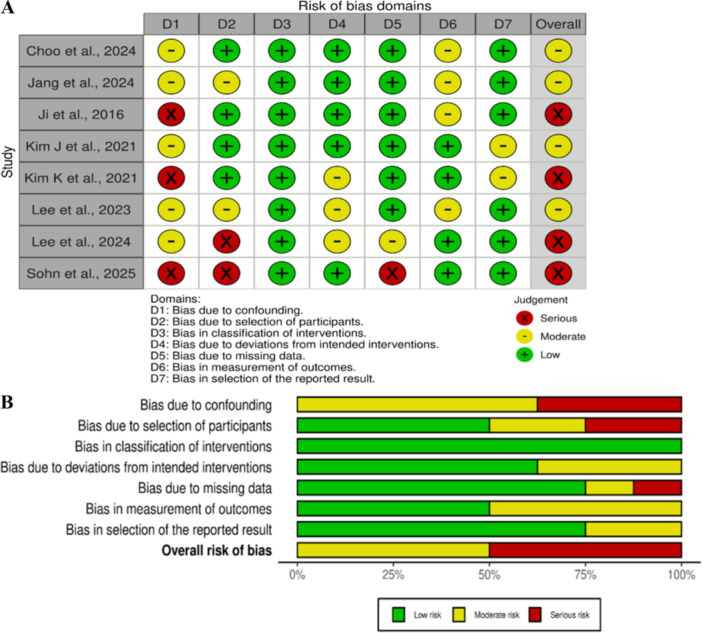
(A) Risk of bias assessment for non‐randomized studies. (B) Summary of risk of bias assessment for non‐randomized studies.

Among the non‐randomized studies, methodological quality varied across domains (Figure 2). Most studies demonstrated low RoB in the classification of interventions and measurement of outcomes. However, notable concerns were observed in domains related to confounding, participant selection, and missing data. Several studies were judged to have a serious overall RoB, primarily driven by high risk in confounding and/or selection processes. Collectively, these findings indicate moderate heterogeneity in study quality, with potential bias in key domains that may influence the robustness of the pooled estimates. Figure 2A,B depict the Risk of Bias Assessment of Non‐Randomized studies.

**Figure 3:**
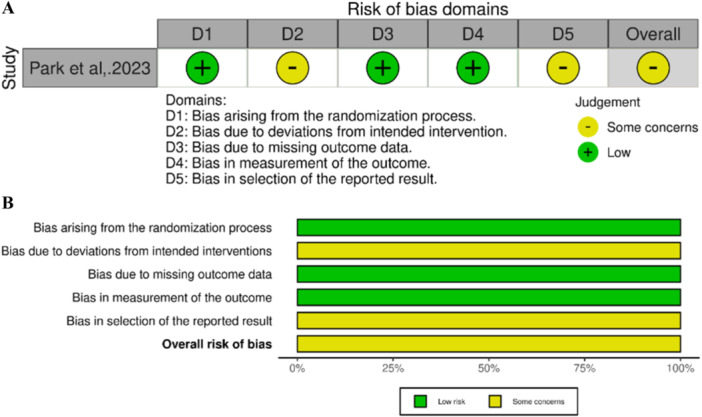
(A) Risk of bias assessment for randomized studies. (B) Summary of risk of bias assessment for randomized studies.

The RoB assessment for the randomized study indicated an overall judgment of some concerns. While domains related to the randomization process, missing outcome data, and outcome measurement were assessed as low risk, concerns were identified in deviations from intended interventions and selective reporting. These limitations suggest a generally acceptable methodological quality, although potential bias in specific domains cannot be excluded (Figure 3A,B).

### 3.3 Outcomes

#### 3.3.1 MACE

##### 3.3.1.1 Overall Analysis

**Figure 4:**
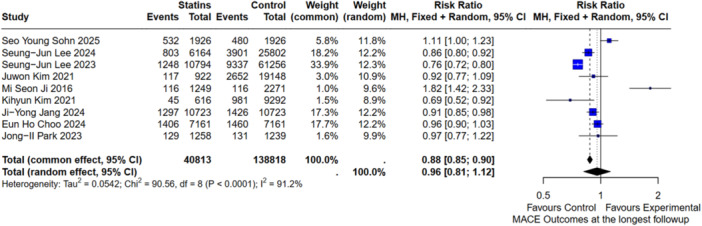
Forest plot showing MACE outcomes at the longest follow‐up.

Nine studies were included in the meta‐analysis for MACE outcomes at the longest follow‐up. The pooled RR under the random‐effects model was 0.96 (95% CI: 0.81–1.12), with substantial heterogeneity (*I* ^2^ = 91.2%; Chi^2^ = 90.56, df = 8, *p* < 0.0001). The CI crossed the null, rendering the overall estimate inconclusive (Figure 4).

##### 3.3.1.2 Influence Analysis (Leave‐One‐Out)

A leave‐one‐out influence analysis was performed to assess the robustness of the pooled estimate. Omission of Mi Seon Ji [26] resulted in a pooled RR of 0.90 (95% CI: 0.82–0.99; *p* = 0.0257), with a reduction in heterogeneity (*I* ^2^ = 87.9%). No other single study meaningfully altered the overall effect size or heterogeneity (Supporting Information S1: Figure 1).

##### 3.3.1.3 Subgroup Analyses

###### 3.3.1.3.1 By Follow‐Up Duration

**Figure 5:**
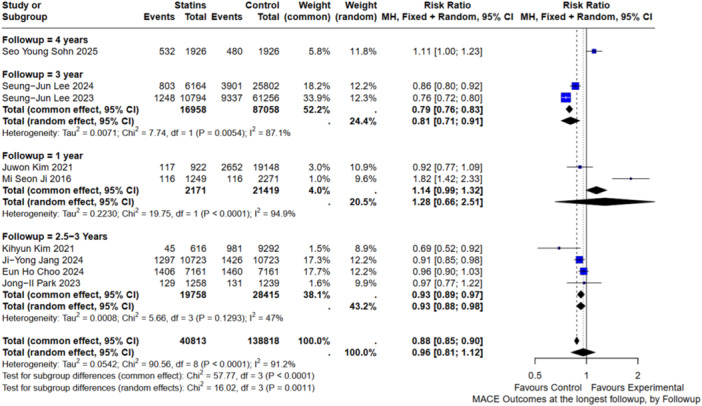
Subgroup analysis of the MACE outcomes by follow‐up length.

Stratification by follow‐up duration revealed statistically significant subgroup differences (random‐effects: Chi^2^ = 16.02, df = 3, *p* = 0.0011). The 2.5–3 years subgroup showed the lowest within‐group heterogeneity (*I* ^2^ = 47.0%) and a significant effect favoring statins (RR 0.93; 95% CI: 0.88–0.98). Results are shown in Figure 5.

###### 3.3.1.3.2 By Study Design

**Figure 6:**
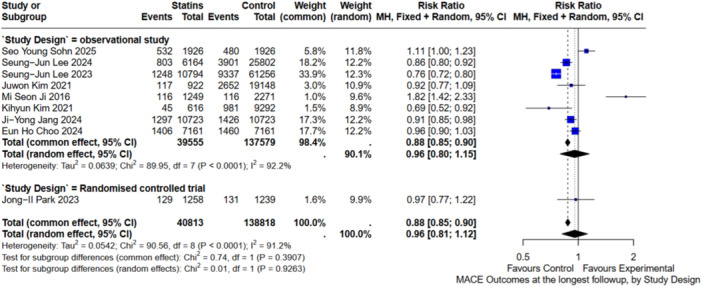
Subgroup analysis of the MACE outcomes by study design.

Only one study was classified as an RCT. Subgroup differences were not significant under the random‐effects model with (total RR 0.96; 95% CI: 0.81−1.12) and (Chi^2^ = 0.01, df = 1, *p* = 0.9263), as detailed in Figure 6. As the subgroup for the RCT had only one study, the analysis was inconclusive.

###### 3.3.1.3.3 By Population (ACS vs. Non‐ACS)

**Figure 7:**
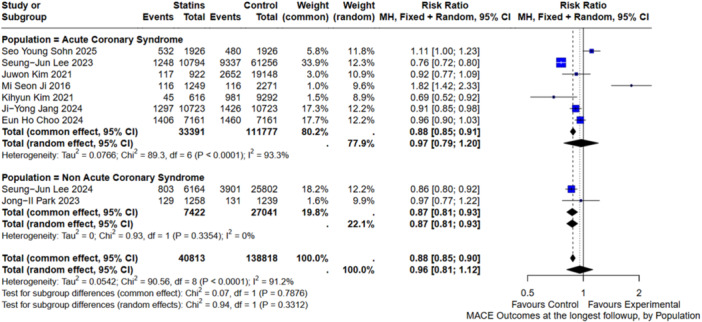
Subgroup analysis of the MACE outcomes by population.

Subgroup differences were not statistically significant (random effects: Chi^2^ = 0.94, df = 1, *p* = 0.3312). Notably, within‐group heterogeneity was eliminated in the non‐ACS subgroup (*I* ^2^ = 0%), with a pooled RR of 0.87 (95% CI: 0.81–0.93) (Figure 7).

###### 3.3.1.3.4 By Type of Statin

**Figure 8:**
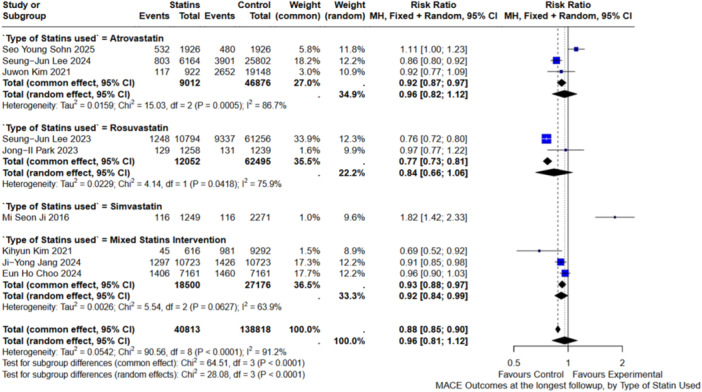
Subgroup analysis of the MACE outcomes by type of statins used.

Significant subgroup differences were observed with a (total RR 0.96; 95% CI 0.81–1.12) and (random‐effects: Chi^2^ = 28.08, df = 3, *p* < 0.0001). Heterogeneity remained high within most subgroups, except for the mixed statins subgroup (*I* ^2^ = 63.9%, *p* = 0.0627). The simvastatin‐only study (Mi Seon Ji [26]) showed a significantly increased risk (RR 1.82; 95% CI: 1.42–2.33), driving much of the observed heterogeneity (Figure 8).

###### 3.3.1.3.5 By RoB

**Figure 9:**
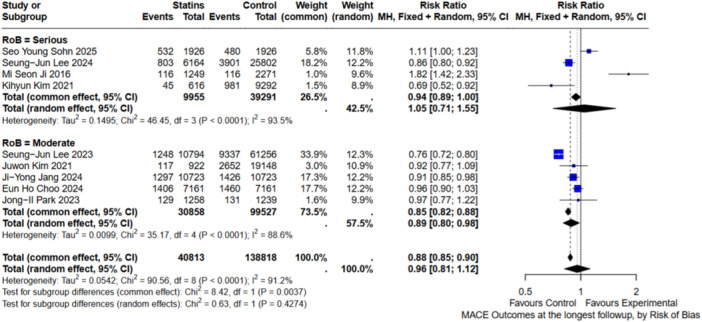
Subgroup analysis of the MACE outcomes by risk of bias.

Under the random‐effects model, subgroup differences were not significant (Chi^2^ = 0.63, df = 1, *p* = 0.4274). However, the “Serious RoB” subgroup showed a pooled RR of 1.05 (95% CI: 0.71–1.55) with very high heterogeneity (*I* ^2^ = 93.5%), while the “Moderate RoB” subgroup yielded a protective effect (RR 0.89; 95% CI: 0.80–0.98; *I* ^2^ = 88.6%) (Figure 9).

#### 3.3.2 All‐Cause Death (ACD) Outcomes

##### 3.3.2.1 Overall Analysis

**Figure 10:**
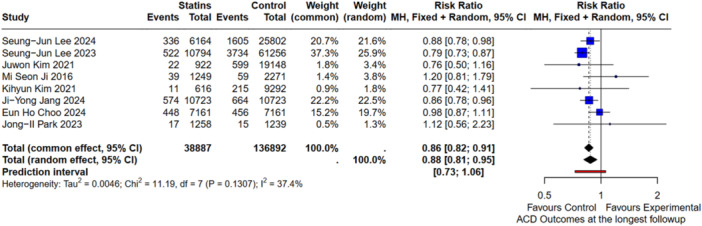
Forest plot showing the ACD outcomes at the longest follow‐up.

Eight studies were included in the meta‐analysis for ACD outcomes at the longest follow‐up. Under the random‐effects model, the pooled RR was 0.88 (95% CI: 0.81–0.95), indicating a statistically significant 12% reduction in all‐cause mortality associated with statin therapy. The 95% prediction interval (PI) ranged from 0.73 to 1.06, suggesting that the true effect in a future study may cross unity. Heterogeneity was moderate (*I* ^2^ = 37.4%; Chi^2^ = 11.19, df = 7, *p* = 0.1307), supporting the overall reliability of the pooled estimate (Figure 10).

###### 3.3.2.1.1 Influence Analysis (Leave‐One‐Out)

A leave‐one‐out sensitivity analysis was performed to assess the robustness of the findings (Supporting Information S1: Figure 2). The pooled effect remained stable across all study omissions (RR range: 0.85–0.91). Omitting Seung‐Jun Lee [23] reduced heterogeneity to zero (*I* ^2^ = 0.0%) with a pooled RR of 0.91 (95% CI: 0.84–0.97). Omission of Eun Ho Choo [28] produced the lowest RR (0.85; 95% CI: 0.79–0.90) and substantially reduced heterogeneity (*I* ^2^ = 6.3%). Removal of Mi Seon Ji [26] slightly lowered the effect estimate to 0.87 (95% CI: 0.80–0.94). Overall, no single study unduly influenced the pooled effect.

##### 3.3.2.2 Subgroup Analyses

###### 3.3.2.2.1 By Follow‐Up Duration

**Figure 11:**
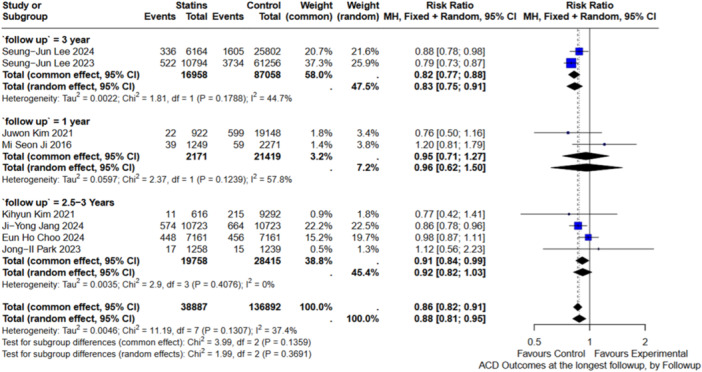
Subgroup analysis of the ACD outcomes by follow‐up length.

Stratification by follow‐up length (Figure 11) revealed no statistically significant between‐subgroup differences (random effects: Chi^2^ = 1.99, df = 2, *p* = 0.3691). The 2.5–3 years subgroup demonstrated zero within‐subgroup heterogeneity (*I* ^2^ = 0.0%, *p* = 0.4076), though the pooled effect was no longer statistically significant (RR 0.92; 95% CI: 0.82–1.03).

###### 3.3.2.2.2 By Study Design

**Figure 12:**
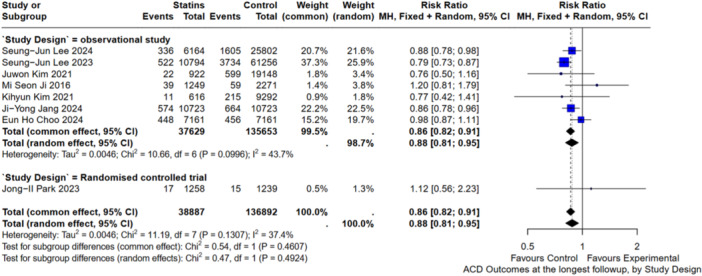
Subgroup analysis of ACD outcomes by study design.

When stratified by study design (Figure 12), between‐subgroup differences were not significant (random‐effects: Chi^2^ = 0.47, df = 1, *p* = 0.4924). The single RCT showed a nonsignificant increased risk (RR 1.12; 95% CI 0.56–2.23); the CI was very wide, reflecting low precision.

###### 3.3.2.2.3 By Population (ACS vs. Non‐ACS)

**Figure 13:**
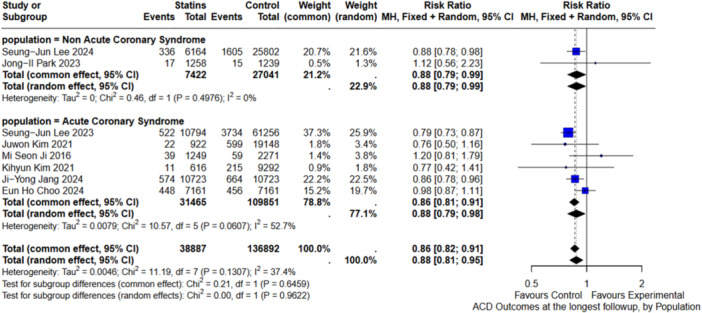
Subgroup analysis of ACD outcomes by population.

No significant between‐subgroup differences were observed (RR 0.88; 95% CI 0.81−0.95) and (random‐effects: Chi^2^ = 0.00, df = 1, *p* = 0.9622). Both subgroups produced identical pooled RRs (RR 0.88). The non‐ACS subgroup showed zero heterogeneity (*I* ^2^ = 0.0%), while the ACS subgroup retained moderate heterogeneity (*I* ^2^ = 52.7%). The non‐ACS subgroup contained only two studies with a notable weight disparity (Figure 13).

###### 3.3.2.2.4 By Type of Statin Used

**Figure 14:**
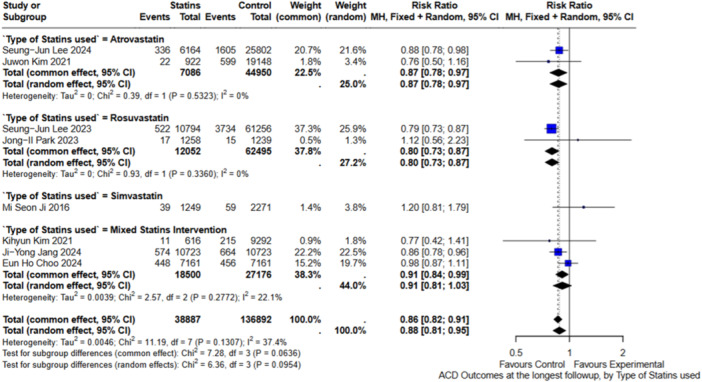
Subgroup analysis of ACD outcomes by type of statins used.

Stratification by statin type (Figure 14) did not yield statistically significant between‐subgroup differences, though the *p* value approached borderline significance (random‐effects: Chi^2^ = 6.36, df = 3, *p* = 0.0954). All subgroups with more than one study demonstrated zero or low heterogeneity (atorvastatin and rosuvastatin: *I* ^2^ = 0.0%; mixed statins: *I* ^2^ = 22.1%). However, each subgroup contained a very small number of studies, limiting interpretability. The simvastatin‐only study (Mi Seon Ji [26]) showed a nonsignificant increased risk (RR 1.20; 95% CI 0.81−1.79).

###### 3.3.2.2.5 By RoB

**Figure 15:**
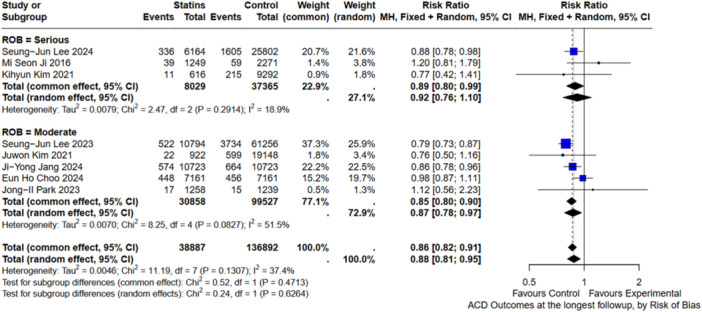
Subgroup analysis of ACD outcomes by risk of bias.

Grouping studies by RoB (moderate vs. serious) (Figure 15) did not yield statistically significant between‐subgroup differences (RR 0.88; 95% CI 0.81–0.95) and (random‐effects: Chi^2^ = 0.24, df = 1, *p* = 0.6264). The serious risk‐of‐bias subgroup yielded a slightly higher (less protective) RR of 0.92 (95% CI: 0.76–1.10) compared to the moderate risk‐of‐bias subgroup (RR 0.87; 95% CI: 0.78–0.97), though the CIs overlapped considerably. Between‐subgroup differences did not approach statistical significance (*p* = 0.6264).

#### 3.3.3 Cardiovascular Death Outcomes

##### 3.3.3.1 Overall Analysis

**Figure 16:**
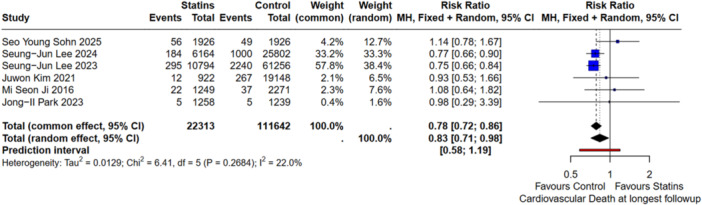
Forest plot showing the meta‐analysis for cardiovascular death at the longest follow‐up.

Six studies were included in the meta‐analysis for cardiovascular death at the longest reported follow‐up (Figure 16). Under the random‐effects model, the pooled RR was 0.83 (95% CI: 0.71–0.98), indicating a statistically significant 17% relative risk reduction in cardiovascular death associated with statin therapy. The 95% PI ranged from 0.58 to 1.19, suggesting that the true effect in a future study may cross the null. Heterogeneity was low (*I* ^2^ = 22.0%; Chi^2^ = 6.41, df = 5, *p* = 0.268), supporting the consistency of the pooled estimate.

##### 3.3.3.2 Influence Analysis (Leave‐One‐Out)

A leave‐one‐out sensitivity analysis was performed to assess the stability of the pooled effect and heterogeneity (Supporting Information S1: Figure 3). Omission of Seo Young Sohn 2025 reduced heterogeneity to zero (*I* ^2^ = 0.0%) and lowered the pooled RR to 0.77 (95% CI: 0.70–0.84), identifying this study as the most influential in the analysis. Removal of Mi Seon Ji [26] similarly reduced heterogeneity to 19.1% and lowered the RR to 0.78 (95% CI: 0.71–0.85). Omission of either Lee 2024 or Lee 2023 attenuated the effect estimate toward the null (RR 0.91 in both cases), rendering it nonsignificant.

##### 3.3.3.3 Subgroup Analyses

###### 3.3.3.3.1 By Follow‐Up Duration

**Figure 17:**
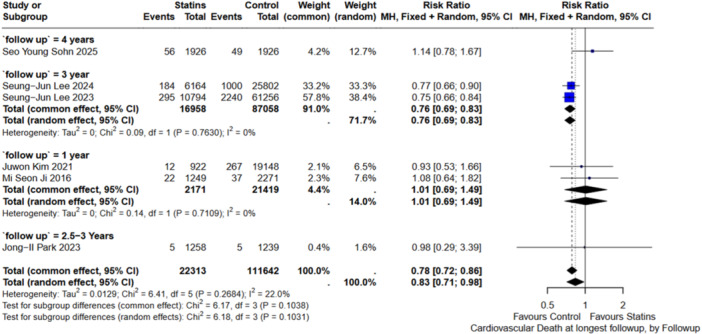
Subgroup analysis of cardiovascular deaths by follow‐up.

Stratification by follow‐up length (Figure 17) revealed no statistically significant between‐subgroup differences (random‐effects: Chi^2^ = 6.18, df = 3, *p* = 0.103). The 3‐year follow‐up subgroup showed zero heterogeneity (*I* ^2^ = 0.0%; *p* = 0.763) with a statistically significant pooled RR of 0.76 (95% CI: 0.69–0.83). The 1‐year subgroup also showed zero heterogeneity but a null effect (RR 1.01; 95% CI: 0.69–1.49). The test for subgroup differences was not significant (*p* = 0.103).

###### 3.3.3.3.2 By Study Design

**Figure 18:**
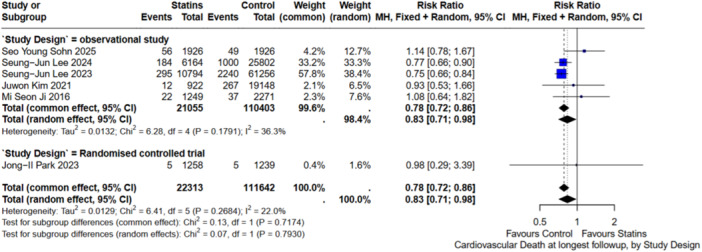
Subgroup analysis of cardiovascular deaths by study design.

When stratified by study design (Figure 18), between‐subgroup differences were not statistically significant (RR 0.83; 95% CI 0.71–0.98) and (random‐effects: Chi^2^ = 0.07, df = 1, *p* = 0.793). The single RCT (Jong‐Il Park [29]) showed a nonsignificant RR of 0.98 (95% CI: 0.29–3.39), with very low precision. The test for subgroup differences was not significant (*p* = 0.793).

###### 3.3.3.3.3 By Type of Statin Used

**Figure 19:**
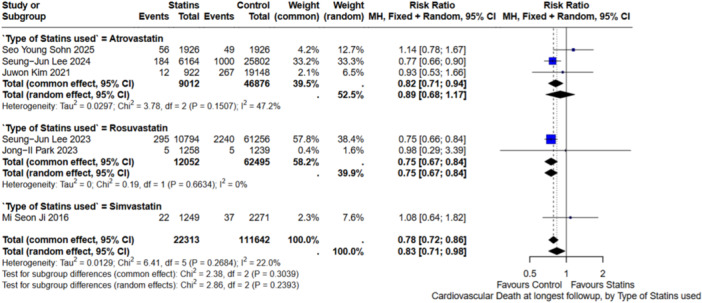
Subgroup analysis of cardiovascular deaths by type of statins used.

Stratification by statin type (Figure 19) did not yield statistically significant between‐subgroup differences (RR 0.83; 95% CI 0.71–0.98) and (random‐effects: Chi^2^ = 2.86, df = 2, *p* = 0.239). The rosuvastatin subgroup demonstrated zero heterogeneity (*I* ^2^ = 0.0%; *p* = 0.663) and a statistically significant pooled RR of 0.75 (95% CI: 0.67–0.84). The atorvastatin subgroup showed moderate heterogeneity (*I* ^2^ = 47.2%) and a nonsignificant pooled RR of 0.89 (95% CI: 0.68–1.17). The test for subgroup differences was not significant (*p* = 0.239).

###### 3.3.3.3.4 By Population (ACS vs. Non‐ACS)

**Figure 20:**
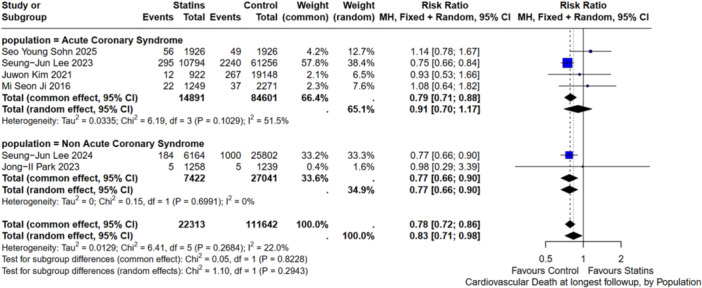
Subgroup analysis of cardiovascular deaths by population.

When studies were divided by population type (Figure 20), between‐subgroup differences did not reach statistical significance (RR 0.83; 95% CI 0.71−0.98) and (random‐effects: Chi^2^ = 1.10, df = 1, *p* = 0.294). The non‐ACS subgroup showed zero heterogeneity (*I* ^2^ = 0.0%; *p* = 0.699) and a statistically significant pooled RR of 0.77 (95% CI: 0.66–0.90). The ACS subgroup showed moderate heterogeneity (*I* ^2^ = 51.5%) and a nonsignificant pooled RR of 0.91 (95% CI: 0.70–1.17). The test for subgroup differences was not significant (*p* = 0.294).

###### 3.3.3.3.5 By RoB

**Figure 21:**
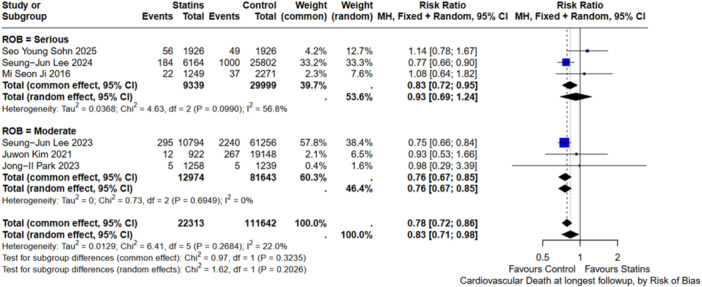
Subgroup analysis of cardiovascular deaths by risk of bias.

Grouping studies by RoB (moderate vs. serious) (Figure 21) resulted in statistically insignificant between‐subgroup differences (random‐effects: Chi^2^ = 1.62, df = 1, *p* = 0.203). The moderate risk‐of‐bias subgroup showed zero heterogeneity (*I* ^2^ = 0.0%; *p* = 0.695) and a statistically significant pooled RR of 0.76 (95% CI: 0.67–0.85). The serious risk‐of‐bias subgroup showed moderate‐to‐high heterogeneity (*I* ^2^ = 56.8%) and a nonsignificant pooled RR of 0.93 (95% CI: 0.69–1.24). The test for subgroup differences was not significant (*p* = 0.203).

#### 3.3.4 Stroke

##### 3.3.4.1 Overall Analysis

**Figure 22:**
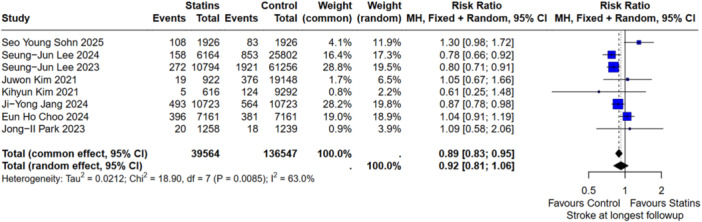
Forest plot showing the pooled RR for stroke at the longest follow‐up.

Eight studies were included in the meta‐analysis for stroke at the longest reported follow‐up (Figure 22). Under the random‐effects model, the pooled RR was 0.92 (95% CI: 0.81–1.06), indicating a nonsignificant 8% relative risk reduction in stroke associated with statin therapy, as the CI crossed the null. Heterogeneity was substantial and statistically significant (*I* ^2^ = 63.0%; Chi^2^ = 18.90, df = 7, *p* = 0.0085), suggesting moderate‐to‐high variability across studies.

##### 3.3.4.2 Influence Analysis (Leave‐One‐Out)

A leave‐one‐out sensitivity analysis was performed to assess the stability of the pooled effect and to identify sources of heterogeneity (Supporting Information S1: Figure 4). Omission of Seo Young Sohn 2025 resulted in the greatest reduction in heterogeneity (to *I* ^2^ = 48.2%) and lowered the pooled RR to 0.88 (95% CI: 0.79–0.99), crossing the threshold of statistical significance (*p* = 0.0263), identifying this study as the most influential in the analysis. Removal of Eun Ho Choo [28] also reduced heterogeneity to 53.9% and lowered the RR to 0.90 (95% CI: 0.77–1.05). Omission of either Lee 2024 or Lee 2023 attenuated the effect estimate toward the null (RR 0.96 in both cases). No other single study meaningfully altered the overall effect size.

##### 3.3.4.3 Subgroup Analyses

###### 3.3.4.3.1 By Follow‐Up Duration

**Figure 23:**
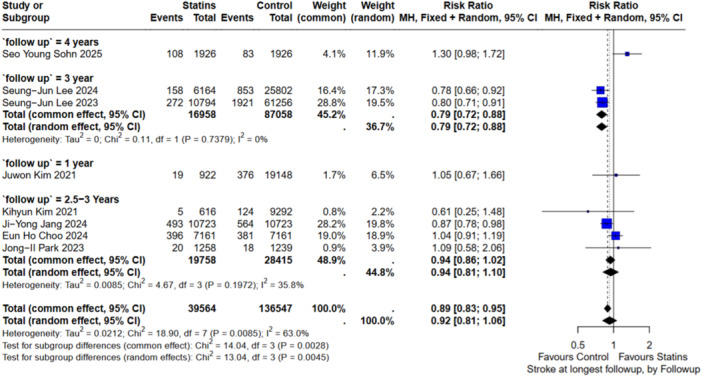
Subgroup analysis for stroke by follow‐up.

Stratification by follow‐up length (Figure 23) revealed statistically significant between‐subgroup differences (common‐effect: Chi^2^ = 14.04, df = 3, *p* = 0.0028; random‐effects: Chi^2^ = 13.04, df = 3, *p* = 0.0045). The 3‐year follow‐up subgroup demonstrated zero heterogeneity (*I* ^2^ = 0.0%; *p* = 0.738) and a statistically significant protective effect (RR 0.79; 95% CI: 0.72–0.88). The 4‐year subgroup (single study) showed a nonsignificant increased risk (RR 1.30; 95% CI: 0.98–1.72). The 2.5–3 years subgroup showed low heterogeneity (*I* ^2^ = 35.8%) and a nonsignificant effect (RR 0.94; 95% CI: 0.81–1.10). Each subgroup suffered from a small sample size, limiting interpretability.

###### 3.3.4.3.2 By Study Design

**Figure 24:**
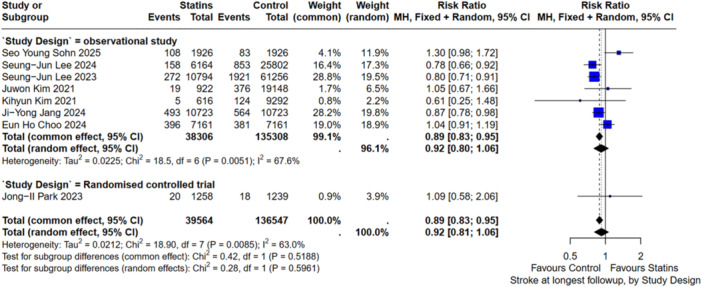
Subgroup analysis for stroke by study design.

When stratified by study design (Figure 24), between‐subgroup differences were not statistically significant (random‐effects: Chi^2^ = 0.28, df = 1, *p* = 0.596). The single RCT (Jong‐Il Park [29]) showed a nonsignificant increased risk (RR 1.09; 95% CI: 0.58–2.06) with very low precision. The test for subgroup differences was not significant (*p* = 0.596).

###### 3.3.4.3.3 By Type of Statin Used

**Figure 25:**
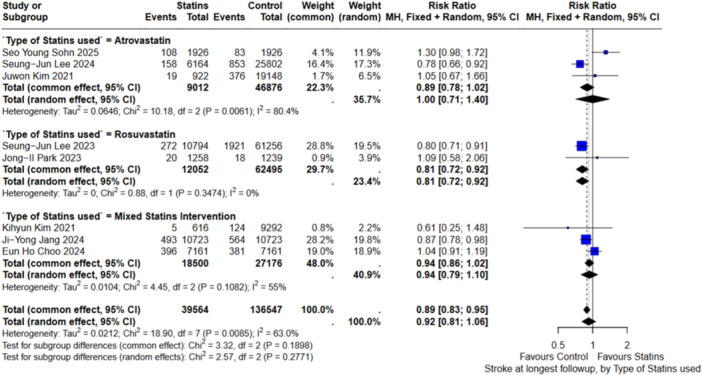
Subgroup analysis for stroke by type of statins used.

Stratification by statin type (Figure 25) did not yield statistically significant between‐subgroup differences (random‐effects: Chi^2^ = 2.57, df = 2, *p* = 0.277). The rosuvastatin subgroup demonstrated zero heterogeneity (*I* ^2^ = 0.0%; *p* = 0.347) and a statistically significant protective effect (RR 0.81; 95% CI: 0.72–0.92). The atorvastatin subgroup showed high heterogeneity (*I* ^2^ = 80.4%) and a null effect (RR 1.00; 95% CI: 0.71–1.40). The mixed statins subgroup showed moderate heterogeneity (*I* ^2^ = 55.0%) and a nonsignificant effect (RR 0.94; 95% CI: 0.79–1.10). The test for subgroup differences was not significant (*p* = 0.277).

###### 3.3.4.3.4 By Population (ACS vs. Non‐ACS)

**Figure 26:**
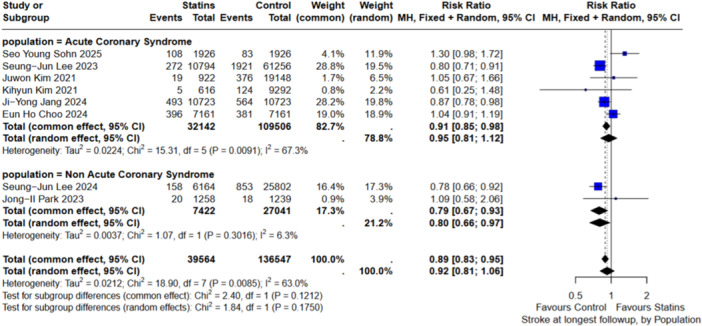
Subgroup analysis for stroke by population.

When studies were divided by population type (Figure 26), between‐subgroup differences were not statistically significant (random effects: Chi^2^ = 1.84, df = 1, *p* = 0.175). The non‐ACS subgroup showed low heterogeneity (*I* ^2^ = 6.3%; *p* = 0.302) and a statistically significant protective effect (RR 0.80; 95% CI: 0.66–0.97). The ACS subgroup showed high heterogeneity (*I* ^2^ = 67.3%*)* and a nonsignificant effect (RR 0.95; 95% CI: 0.81–1.12). The test for subgroup differences was not significant (*p* = 0.175).

###### 3.3.4.3.5 By RoB

**Figure 27:**
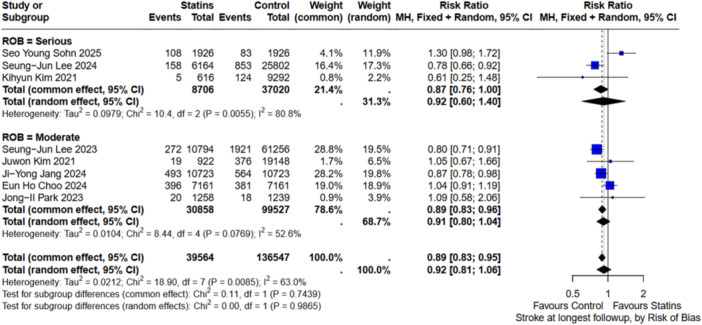
Subgroup analysis for stroke by risk of bias.

Grouping studies by RoB (moderate vs. serious) (Figure 27) resulted in statistically insignificant between‐subgroup differences (random‐effects: Chi^2^ = 0.00, df = 1, *p* = 0.987). The moderate risk‐of‐bias subgroup showed a point estimate approaching statistical significance (RR 0.91; 95% CI: 0.80–1.04) with moderate heterogeneity (*I* ^2^ = 52.6%). The serious risk‐of‐bias subgroup showed high heterogeneity (*I* ^2^ = 80.8%) and a nonsignificant effect (RR 0.92; 95% CI: 0.60–1.40). The test for subgroup differences was not significant (*p* = 0.987).

#### 3.3.5 MI

##### 3.3.5.1 Overall Analysis

**Figure 28:**
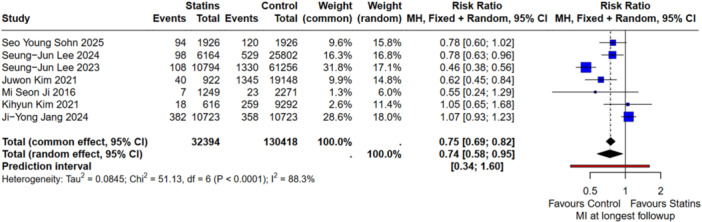
Forest plot showing the meta‐analysis for the RR of MI.

Seven studies were included in the meta‐analysis for MI at the longest reported follow‐up (Figure 28). Under the random‐effects model, the pooled RR was 0.74 (95% CI: 0.58–0.95), indicating a statistically significant 26% relative risk reduction in MI associated with statin therapy. The 95% PI ranged from 0.34 to 1.60, suggesting that the true effect in a future study may cross the null. Heterogeneity was significantly high (*I* ^2^ = 88.3%; Chi^2^ = 51.13, df = 6, *p* < 0.0001), warranting cautious interpretation.

##### 3.3.5.2 Influence Analysis (Leave‐One‐Out)

A leave‐one‐out sensitivity analysis was performed to assess the stability of the pooled effect and to identify sources of heterogeneity (Supporting Information S1: Figure 5). Omission of Seung‐Jun Lee [23] decreased heterogeneity the most (68.6%), while omission of Ji‐Yong Jang [27] had the greatest impact on the pooled effect estimate (*I* ^2^ = 75.8%, RR 0.68; 95% CI 0.53−0.86), identifying these as the most influential studies in the analysis.

##### 3.3.5.3 Subgroup Analyses

###### 3.3.5.3.1 By Follow‐Up Duration

**Figure 29:**
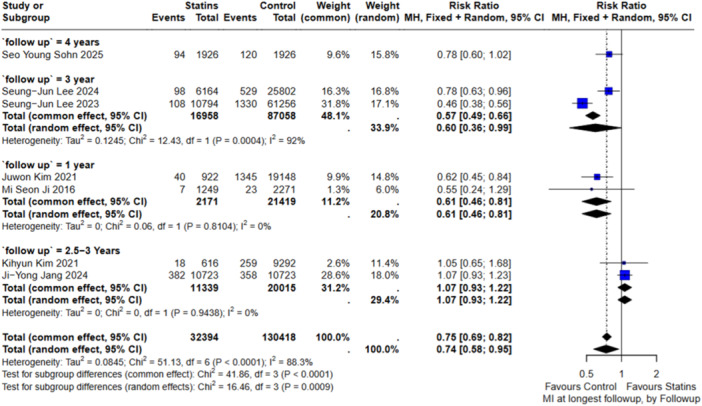
Subgroup analysis of MI at longest follow‐up by length of follow‐up.

The subgroup analysis for MI, stratified by follow‐up duration, revealed statistically significant between‐subgroup differences (Figure 29) (random‐effects: Chi^2^ = 16.46, df = 3, *p* = 0.009), indicating that the effect of statin therapy on MI risk varies significantly depending on the length of follow‐up. The 1‐year follow‐up subgroup demonstrated zero heterogeneity (*I* ^2^ = 0.0%; *p* = 0.810) and a statistically significant protective effect (RR 0.61; 95% CI: 0.46–0.81). The 3‐year subgroup showed the largest protective effect (RR 0.60; 95% CI: 0.36–0.99) but with very high heterogeneity (*I* ^2^ = 92.0%). The 2.5–3 years subgroup showed a null effect with zero heterogeneity (RR 1.07; 95% CI: 0.93–1.22). The test for subgroup differences was statistically significant (*p* = 0.0009).

###### 3.3.5.3.2 By Type of Statin Used

**Figure 30:**
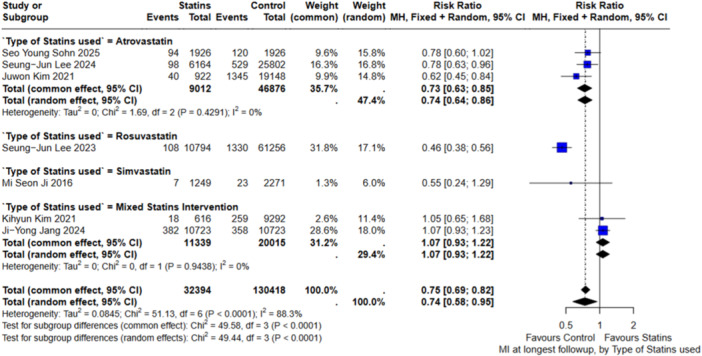
Subgroup analysis of MI at longest follow‐up by type of statins used.

Stratification by statin type (Figure 30) yielded statistically significant between‐subgroup differences (random‐effects: Chi^2^ = 49.44, df = 3, *p* < 0.0001). The atorvastatin subgroup demonstrated zero heterogeneity (*I* ^2^ = 0.0%; *p* = 0.429) and a statistically significant protective effect (RR 0.74; 95% CI: 0.64–0.86), representing a 27% reduction in MI risk. The rosuvastatin subgroup (single study: Seung‐Jun Lee [23]) showed the largest protective effect (RR 0.46; 95% CI: 0.38–0.56), a 54% reduction in MI risk. The simvastatin subgroup (single study: Mi Seon Ji [26]) showed a nonsignificant protective effect (RR 0.55; 95% CI: 0.24–1.29), with a wide CI crossing the null. The mixed statins subgroup showed a null effect (RR 1.07; 95% CI: 0.93–1.22) with zero heterogeneity (*I* ^2^ = 0.0%; *p* = 0.944), indicating that in studies using mixed statin interventions, no reduction in MI risk was observed. The test for subgroup differences was highly statistically significant (*p* < 0.0001 under both common‐effect and random‐effects models), confirming that the type of statin used is a significant effect modifier for MI outcomes.

###### 3.3.5.3.3 By Population (ACS vs. Non‐ACS)

**Figure 31:**
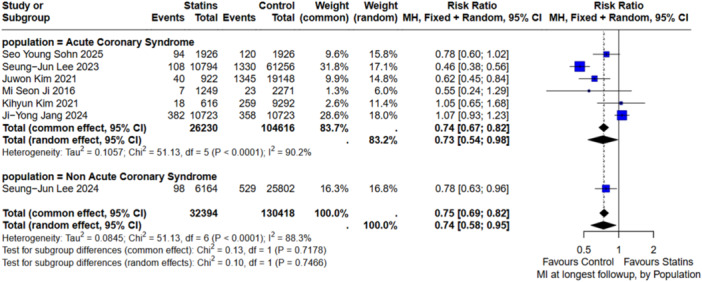
Subgroup analysis of MI at longest follow‐up by population.

When studies were divided by population type (Figure 31), between‐subgroup differences did not reach statistical significance (random effects: Chi^2^ = 0.10, df = 1, *p* = 0.747). The ACS subgroup showed a statistically significant protective effect (RR 0.73; 95% CI: 0.54–0.98), representing a 26% reduction in MI risk. However, heterogeneity within this subgroup was very high (*I* ^2^ = 90.2%; *p* < 0.0001), indicating substantial variability across the six studies. The non‐ACS subgroup consisted of a single study, which showed a significant protective effect (RR 0.78; 95% CI: 0.63–0.96), representing a 22% reduction in MI risk. The test for subgroup differences was not statistically significant (common‐effect: *p* = 0.718; random‐effects: *p* = 0.747), indicating that the effect of statin therapy on MI risk does not differ significantly between ACS and non‐ACS populations.

###### 3.3.5.3.4 By RoB

**Figure 32:**
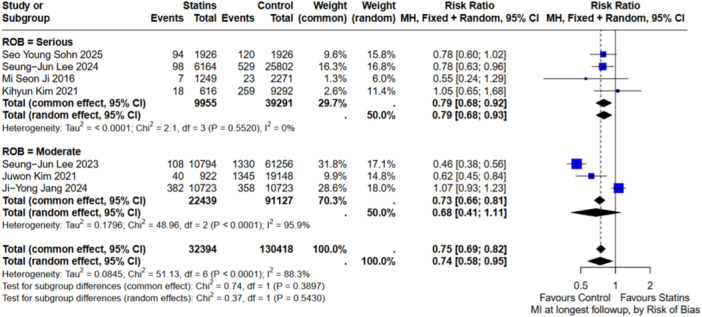
Subgroup analysis of MI at longest follow‐up by risk of bias.

Grouping studies by RoB (Figure 32) resulted in statistically insignificant between‐subgroup differences (random‐effects: Chi^2^ = 0.37, df = 1, *p* = 0.543). The serious RoB subgroup demonstrated zero heterogeneity (*I* ^2^ = 0.0%; *p* = 0.552) and a statistically significant protective effect (RR 0.79; 95% CI: 0.68–0.93), representing a 21% reduction in MI risk. The consistency across these studies is excellent.

The moderate RoB subgroup showed a larger protective effect (RR 0.73; 95% CI: 0.66–0.81). However, heterogeneity within this subgroup was extremely high (*I* ^2^ = 95.9%; *p* < 0.0001), indicating substantial variability across the three studies. This heterogeneity is likely driven by the divergent effects observed in this subgroup: Seung‐Jun Lee [23] (RR 0.46), Juwon Kim 2021 (RR 0.62), and Ji‐Yong Jang [27] (RR 1.07).

The test for subgroup differences was not statistically significant (common‐effect: *p* = 0.390; random‐effects: *p* = 0.543), indicating that the effect of statin therapy on MI risk did not differ significantly between studies classified as having serious versus moderate RoB.

#### 3.3.6 Revascularization

##### 3.3.6.1 Overall Analysis

**Figure 33:**
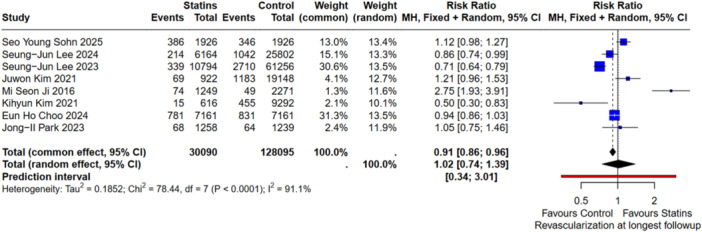
Forest plot showing the meta‐analysis for revascularization at the longest follow‐up.

Eight studies were included in the meta‐analysis for revascularization at the longest reported follow‐up (Figure 33). Under the random‐effects model, the pooled RR was 1.02 (95% CI: 0.74–1.39), indicating a null effect, as the CI crossed unity. The 95% PI ranged from 0.34 to 3.01, reflecting substantial uncertainty about the true effect in future studies. Heterogeneity was significant and exceptionally high (*I* ^2^ = 91.1%; Chi^2^ = 78.44, df = 7, *p* < 0.0001), suggesting very poor consistency across studies and warranting extremely cautious interpretation.

##### 3.3.6.2 Influence Analysis (Leave‐One‐Out)

A leave‐one‐out sensitivity analysis was performed to assess the stability of the pooled effect and to identify sources of heterogeneity (Supporting Information S1: Figure 6). Omission of Mi Seon Ji [26] resulted in the greatest reduction in heterogeneity (from *I* ^2^ = 91.1% to 85.5%) and produced the largest change in the pooled effect estimate, lowering the RR to 0.91 (95% CI: 0.76–1.08; *p* = 0.289). Although the CI remained wide, this omission moved the point estimate toward a protective effect (9% reduction) and substantially reduced heterogeneity.

Omission of Seung‐Jun Lee [23] also notably reduced heterogeneity to *I* ^2^ = 87.9%, though the pooled RR increased to 1.08 (95% CI: 0.76–1.51). Omission of Kihyun Kim [25] produced the highest pooled RR (1.10; 95% CI: 0.81–1.48), suggesting that this study had a dampening effect on the overall estimate.

No other single study meaningfully altered the overall effect size or heterogeneity to a comparable degree as Mi Seon Ji [26]. The CIs for all leave‐one‐out estimates crossed the null, consistent with the original analysis.

##### 3.3.6.3 Subgroup Analyses

###### 3.3.6.3.1 By Study Design

**Figure 34:**
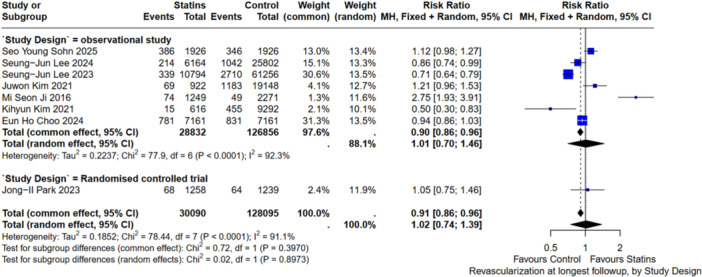
Subgroup analysis of revascularization by study design.

When stratified by study design (Figure 34), between‐subgroup differences were not statistically significant (random‐effects: Chi^2^ = 0.02, df = 1, *p* = 0.897). The observational study subgroup showed a null effect (RR 1.01; 95% CI: 0.70–1.46) with very high heterogeneity (*I* ^2^ = 92.3%; *p* < 0.0001), indicating substantial inconsistency across the seven observational studies.

The RCT subgroup consisted of a single study (Jong‐Il Park [29]), which showed a null effect (RR 1.05; 95% CI: 0.75–1.46), consistent with the overall findings.

The test for subgroup differences was not statistically significant (common‐effect: *p* = 0.397; random‐effects: *p* = 0.897), indicating that the effect of statin therapy on revascularization did not differ significantly between observational studies and RCTs.

###### 3.3.6.3.2 By Follow‐Up Duration

**Figure 35:**
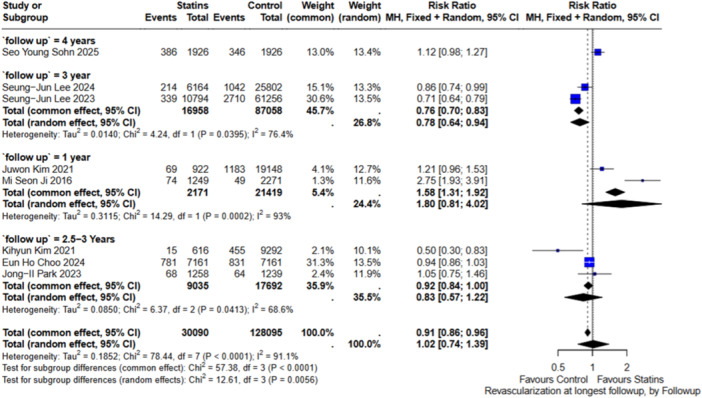
Subgroup analysis of revascularization by length of follow‐up.

Stratification by follow‐up length (Figure 35) revealed statistically significant between‐subgroup differences (random‐effects: Chi^2^ = 12.61, df = 3, *p* = 0.0056). The 3‐year follow‐up subgroup showed a statistically significant protective effect (RR 0.78; 95% CI: 0.64–0.94). However, heterogeneity was high (*I* ^2^ = 76.4%; *p* = 0.040). The 1‐year follow‐up subgroup showed a statistically significant harmful effect (RR 1.58; 95% CI: 1.31–1.92). Heterogeneity was very high (*I* ^2^ = 93.0%; *p* = 0.0002). The 2.5–3 years follow‐up subgroup showed a borderline protective effect (RR 0.92; 95% CI: 0.84–1.00), with moderate heterogeneity (*I* ^2^ = 68.6%; *p* = 0.041). The 4‐year follow‐up subgroup (single study: Seo Young Sohn 2025) showed a borderline increased risk (RR 1.12; 95% CI: 0.98–1.27). The test for subgroup differences was statistically significant (random‐effects: *p* = 0.0056), confirming that follow‐up duration is a significant effect modifier for revascularization outcomes.

###### 3.3.6.3.3 By Population (ACS vs. Non‐ACS)

**Figure 36:**
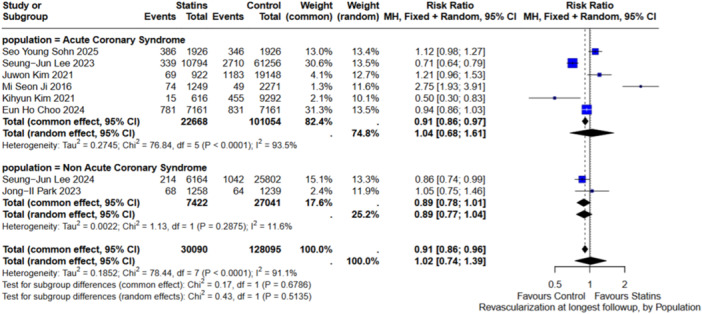
Subgroup analysis of revascularization by population.

When studies were grouped by population (Figure 36), between‐subgroup differences did not reach statistical significance (random‐effects: Chi^2^ = 0.43, df = 1, *p* = 0.514). The ACS subgroup showed a null effect (RR 1.04; 95% CI: 0.68–1.61) with very high heterogeneity (*I* ^2^ = 93.5%*; p* < 0.0001), indicating substantial inconsistency across the six studies in ACS populations. The non‐ACS subgroup showed a borderline protective effect (RR 0.89; 95% CI: 0.78–1.01; *p* = 0.07) with low heterogeneity (*I* ^2^ = 11.6%; *p* = 0.288), suggesting consistent findings across these two studies. The test for subgroup differences was not statistically significant (common‐effect: *p* = 0.679; random‐effects: *p* = 0.514), indicating that the effect of statin therapy on revascularization did not differ significantly between ACS and non‐ACS populations.

###### 3.3.6.3.4 By Type of Statin Used

**Figure 37:**
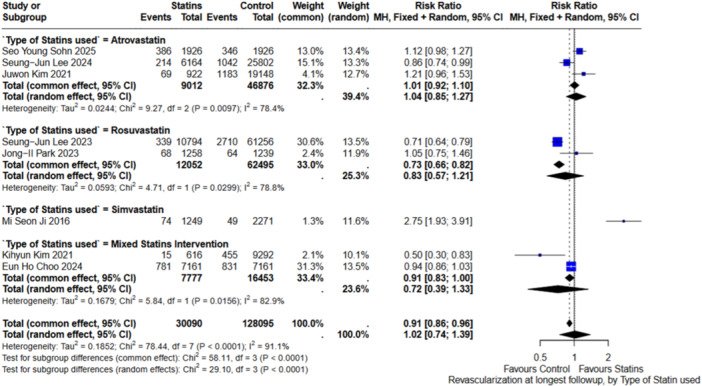
Subgroup analysis of revascularization by type of statins used.

Stratification by statin type (Figure 37) yielded statistically significant between‐subgroup differences (random‐effects: Chi^2^ = 29.10, df = 3, *p* < 0.0001). The atorvastatin subgroup showed a null effect (RR 1.04; 95% CI: 0.85–1.27) with high heterogeneity (*I* ^2^ = 78.4%). The rosuvastatin subgroup showed a statistically significant protective effect (RR 0.83; 95% CI: 0.57–1.21); heterogeneity was high (*I* ^2^ = 78.8%). The simvastatin subgroup (single study: Mi Seon Ji [26]) showed a statistically significant harmful effect (RR 2.75; 95% CI: 1.93–3.91). This study was also identified as the most influential in the leave‐one‐out analysis. The mixed statins subgroup showed a null effect (RR 0.72; 95% CI: 0.39–1.33) with very high heterogeneity (*I* ^2^ = 82.9%). The test for subgroup differences was highly statistically significant (random‐effects: *p* < 0.0001), confirming that the type of statin used is a significant effect modifier for revascularization outcomes.

###### 3.3.6.3.5 By RoB

**Figure 38:**
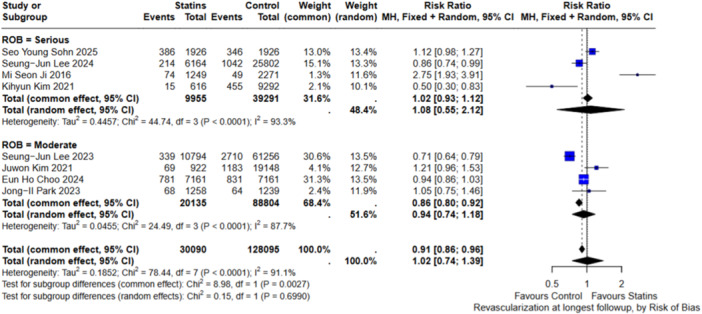
Subgroup analysis of revascularization by risk of bias.

When studies were divided by RoB (Figure 38), between‐subgroup differences were not statistically significant under the random‐effects model (random‐effects: Chi^2^ = 0.15, df = 1, *p* = 0.699). The serious RoB subgroup showed a null effect (RR 1.08; 95% CI: 0.55–2.12) with very high heterogeneity (*I*2 = 93.3%; *p* < 0.0001). The moderate RoB subgroup also showed a null effect (RR 0.94; 95% CI: 0.74–1.18) with very high heterogeneity (*I* ^2^ = 87.7%; *p* < 0.0001). The test for subgroup differences was not statistically significant under the random‐effects model (common‐effect: *p* = 0.003; random‐effects: *p* = 0.699).

#### 3.3.7 Rhabdomyolysis

##### 3.3.7.1 Overall Analysis

**Figure 39:**
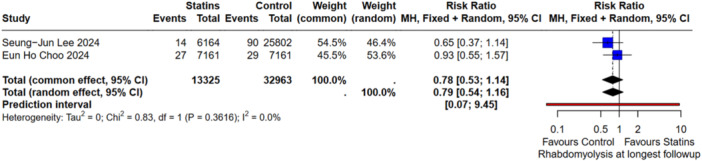
Forest plot showing the meta‐analysis for rhabdomyolysis at the longest follow‐up.

Two studies were included in the meta‐analysis for rhabdomyolysis at the longest reported follow‐up (Figure 39). Under the random‐effects model, the pooled RR was 0.79 (95% CI: 0.54–1.16), indicating a nonsignificant 21% relative risk reduction in rhabdomyolysis associated with statin therapy, as the CI crossed the null. The 95% PI ranged from 0.07 to 9.45, reflecting extreme uncertainty due to the small number of studies. Heterogeneity was zero (*I* ^2^ = 0.0%; Chi^2^ = 0.83, df = 1, *p* = 0.362), indicating perfect consistency between the two included studies.

##### 3.3.7.2 New Onset Diabetes Mellitus

###### 3.3.7.2.1 Overall Analysis

**Figure 40:**
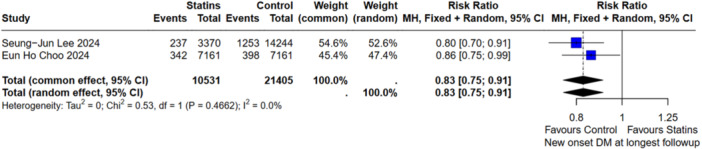
Forest plot showing the meta‐analysis for new‐onset DM at the longest follow‐up.

Two studies were included in the meta‐analysis for new‐onset diabetes mellitus (DM) at the longest reported follow‐up (Figure 40). Under the random‐effects model, the pooled RR was 0.83 (95% CI: 0.75–0.91), indicating a statistically significant 17% relative risk reduction in new‐onset DM associated with statin therapy. Heterogeneity was zero (*I* ^2^ = 0.0%; Chi^2^ = 0.53, df = 1, *p* = 0.466), indicating perfect consistency between the two included studies.

## 4 Discussion

In this systematic review and meta‐analysis, including 179 621 subjects undergoing PCI, we compared the efficacy of intensive lipid‐lowering therapy consisting of moderate‐intensity statins plus ezetimibe versus high‐intensity statins alone. No significant difference was found in overall cardiovascular outcomes represented by MACE, whereas nominal (non‐robust) and heterogeneity‐sensitive differences were observed for some specific cardiovascular endpoints like cardiovascular death, MI, and stroke, indicating their limited precision and uncertain generalizability.

### 4.1 Main Findings and Clinical Significance

According to the main comparison, there was no significant difference between the two regimens concerning MACE (RR 0.96, 95% CI 0.81–1.12), with the wide heterogeneity among trials. Hence, the results show that in the population after PCI, regardless of whether an additional lipid‐lowering strategy (ezetimibe in this case) was used or not, there would be similar cardiovascular benefits, at least at the group level. Importantly, the equivalence does not suggest any therapeutic redundancy but may reflect possible similarity in the degree of lowering of LDL‐C level by two different means.

The secondary analyses of MACE components have shown a statistically non‐robust signal toward cardiovascular mortality reduction (RR 0.83, 95% CI 0.71–0.98) and a nominal reduction in MI rate (RR 0.74, 95% CI 0.58–0.95). Again, it is worth noting that the risk estimates were characterized by high heterogeneity and broad PI, which could affect their credibility.

On the contrary, the results for revascularization were not in favor of the experimental strategy (RR 1.02, 95% CI 0.74–1.39). Thus, it can be seen that in most cases, the risk of reintervention is determined by the specifics of the procedure and the patient's condition rather than lipid‐lowering strategy.

### 4.2 Interpretation of Subgroup Analyses

Subgroup analyses provide relevant mechanistic and methodological insights but need to be interpreted cautiously because of problems with multiplicity and residual confounding. In addition, subgroup findings should be considered exploratory due to limited power and potential interaction instability.

### 4.3 Follow‐Up Duration

Subgroup differences reached statistical significance in relation to the outcomes MACE and MI based on follow‐up duration, with heterogeneity attenuated in the intermediate‐term follow‐up (2.5−3 years). This observation is biologically reasonable since stable plaques require a continuous period of exposure to lipid‐lowering agents, in accordance with previous long‐term trials in this area [16, 30].

### 4.4 Study Design

There is no evidence of statistically significant differences based on the RCT‐observational dichotomy, although the strong preponderance of observational cohorts (eight out of nine studies used) poses a limitation regarding causal interpretation of findings. Biasing toward the null in some analyses is likely from residual confounding by indication, whereby high‐intensity statins were employed preferentially in higher‐risk subjects.

### 4.5 ACS Versus Non‐ACS Populations

No statistically significant subgroup interaction was found between ACS and non‐ACS populations, suggesting comparable relative risks associated with treatments across clinical phenotypes. However, reduced heterogeneity to zero was observed in the non‐ACS population for several outcomes, indicating that clinical stability can act as a modifier of treatment response variability.

### 4.6 Type of Statin Strategy

Interestingly, differences by type of statin strategy were statistically significant for MACE, MI, and revascularization. The fact that “high‐intensity monotherapy” and “moderate‐intensity statin plus ezetimibe” approaches show such divergent responses can only be explained by distinct mechanisms underlying them, taking into account their different degrees of achieved LDL‐C reductions [31, 32, 33].

### 4.7 RoB

Heterogeneity remained unaltered following stratification by RoB in almost all analyses. Despite the lack of statistical significance in some cases, residual heterogeneity persisted in the pooled analyses of both moderate‐risk and serious‐risk strata, consistent with the prevalence of observational design among the included articles. This raises the possibility that unmeasured confounding and detection bias may have influenced the observed associations.

### 4.8 Mechanistic Considerations

Biological plausibility for similar results from combination lipid‐lowering therapy compared with high‐intensity statin monotherapy relies on complementary pathways for LDL‐C reduction. Inhibition of the synthesis of cholesterol in the liver mediated by HMG‐CoA reductase blockade underlies statin action, while inhibition of the intestinal absorption of cholesterol through the inhibition of NPC1L1 characterizes ezetimibe [34, 35, 36]. Hence, this dual approach leads to additional reduction in LDL‐C levels without an increase in pleiotropic effects of the statin itself, including improvements in endothelial function, stability of the plaque, and inflammation.

Nevertheless, whether pleiotropic effects translate into clinically beneficial outcomes besides the reduction in LDL‐C levels has been a subject of controversy. According to recent studies, the magnitude of LDL‐C reduction is the key component in decreasing cardiovascular risk, regardless of the class of medication used [37, 38, 39]. Therefore, this assumption explains the observed similarities between outcomes of combination and monotherapies.

### 4.9 Comparison With Existing Evidence

Current study findings corroborate previous data from large prospective randomized trials indicating that similar outcomes of high‐intensity statin monotherapy are achieved in combination lipid‐lowering treatment, provided that a similar reduction in LDL‐C levels is reached. IMPROVE‐IT trial revealed that adding ezetimibe to the treatment of patients with ACS who received statin therapy resulted in decreased incidence of cardiovascular events [40].

Moreover, meta‐analysis comparing statin therapy, ezetimibe, and PCSK9 inhibitors, among other medications, found that a linear relationship exists between the magnitude of reduction in absolute levels of LDL‐C and cardiovascular risk [38, 41, 42]. However, direct comparison between combination moderate‐intensity statin and high‐intensity statin therapy has not been extensively researched before the current study.

### 4.10 Clinical Implications

The present results suggest that moderate‐intensity statin therapy together with ezetimibe might represent an effective replacement strategy for high‐intensity statin therapies in patients with PCI, especially in cases of statin intolerance, higher risk for adverse effects, and inadequate LDL‐C response. The current guidelines recommend personalized LDL‐C lowering treatment options instead of escalation to maximal statin doses [5, 6].

The marginal benefits seen for cardiovascular mortality and MI in some analyses point to possible clinical significance among select subgroups of patients, although these would have to be confirmed through randomized studies

### 4.11 Limitations

This meta‐analysis has several limitations, the first one being the dominance of observational studies and, consequently, an increased threat of confounding by indication and treatment selection bias. Furthermore, significant heterogeneity among most endpoints makes the pooled estimates less precise. Importantly, heterogeneity also affected the stability of effect estimates under different statistical models (fixed vs. random effects). In addition, variations in statin drugs used, ezetimibe dose, LDL‐C level at the time of study entry, and patient compliance with the prescribed regimen might explain the differences between studies. Additionally, only one RCT was available for inclusion in the meta‐analysis. Moreover, detection bias and residual confounding inherent to administrative and registry‐based data sets cannot be excluded. Definitions and ascertainment of outcomes, such as incident diabetes, varied across studies and were not consistently adjudicated, which may introduce misclassification bias. External validity may also be limited, as a substantial proportion of included studies originated from East Asian registry‐based populations. Differences in baseline cardiovascular risk profiles, statin prescribing patterns, achieved LDL‐C targets, and pharmacogenomic variability may limit generalizability to Western or more ethnically diverse populations. Finally, definitions of MACE and secondary end‐points differed between included studies.

### 4.12 Future Research Directions

RCTs are clearly needed to compare moderate‐intensity statins plus ezetimibe and high‐intensity statins in the post‐PCI population and assess whether any benefits associated with the combination of moderate‐intensity statins and ezetimibe persist. It would be interesting to use stratification according to baseline LDL‐C levels, presence of ACS, and other relevant variables like gene or metabolomics profiles associated with statin responsiveness. Besides, imaging‐based endpoints, such as plaque regression or reduction in atheroma burden, could be applied to investigate the mechanism behind the observed differences.

## 5 Conclusions

The current meta‐analysis has revealed that moderate‐intensity statins together with ezetimibe were associated with similar pooled cardiovascular outcomes compared with high‐intensity statins among patients with PCI, with potential signals of benefit in terms of cardiovascular mortality and MI. However, heterogeneity and the dominant role of observational studies preclude firm causal conclusions and limit confidence in effect estimates. These findings support a more individualized approach to lipid‐lowering therapy post‐PCI, emphasizing LDL‐C reduction as the principal therapeutic target rather than statin intensity alone. However, these results should be interpreted cautiously, given heterogeneity, study design limitations, and potential bias.

## Author Contributions

**Emad Uddin Sajid:** conceptualization, methodology, supervision, validation, writing – review and editing. **Muhammad Hasnain Azeem:** data curation, formal analysis, investigation, writing – original draft, quality assessment. **Alishba Fatima:** methodology, literature search, data curation, writing – original draft, visualization. **Syeda Masooma Jafri:** investigation, data extraction, writing – review and editing. **Danish Hassan:** statistical analysis, software, validation, writing – review and editing. **Taha Ibrahim:** literature search, data curation, investigation. **Kashish Zehra Manjee:** data extraction, writing – review and editing. **Syed Rayyan Ahmed:** formal analysis, interpretation of data, visualization. **Tooba Ali:** investigation, data verification. **Rida Shakeel:** literature review, data curation. **Muhammad Usman:** validation, writing – review and editing. **Raghabendra Kumar Mahato:** supervision, critical revision of the manuscript, writing – review and editing. **Danish Bawa:** critical revision of the manuscript, final approval of the manuscript. All authors reviewed and approved the final version of the manuscript.

## Funding

The authors have nothing to report.

## Ethics Statement

Ethical approval and informed consent were not required for this study because it is a systematic review and meta‐analysis based on previously published studies.

## Conflicts of Interest

The authors declare no conflicts of interest.

## Supporting information

Supporting File

## Data Availability

The data supporting the findings of this study are available within the article and its supplementary materials. No new data sets were generated outside the included published studies.
