# Supplementary material for: Moderate Intensity Statins Plus Ezetimibe Combination Therapy Versus High Intensity Statins Monotherapy After Percutaneous Coronary Intervention: A Systematic Review and Meta‐Analysis
Source: Clin Cardiol. 2026 Jun 15;49(6):e70369. doi: 10.1002/clc.70369 (PMC13267995; doi:10.1002/clc.70369)
Supplement: Supplementary file 1 — Supporting File [file CLC-49-e70369-s001.docx]

**Online Supplementary File**

**INDEX**

| S.NO | Detail | Page No |
| --- | --- | --- |
|  | Search Strategy | 2-4 |
|  | Supplementary Tables | 5-7 |
|  | Supplementary Figures | 7-10 |

**Supplementary Tables**

**Supplementary Table 1:** Detailed search strategies used for each database

| Data Base | Search Strategy | Search Results |
| --- | --- | --- |
| PubMed | moderate intensity statins plus ezetimibe AND high intensity statins AND percutaneous coronary intervention  statins plus ezetimibe AND percutaneous coronary intervention  (Atorvastatin plus Ezetimibe OR Rosuvastatin plus Ezetimibe OR Simvastatin plus Ezetimibe OR Pravastatin plus Ezetimibe OR Lovastatin plus Ezetimibe OR Fluvastatin plus Ezetimibe) AND (Atorvastatin OR Rosuvastatin) AND percutaneous coronary intervention  (moderate-intensity statin OR atorvastatin OR rosuvastatin OR pravastatin OR fluvastatin OR lovastatin) AND (ezetimibe) AND (high-intensity statin OR atorvastatin 40 mg OR rosuvastatin 20 mg) AND (percutaneous coronary intervention OR PCI)  ("moderate-intensity statin" OR "atorvastatin" OR "rosuvastatin" OR "pravastatin" OR "fluvastatin" OR "lovastatin") AND ("ezetimibe") AND ("high-intensity statin" OR "atorvastatin 40 mg" OR "rosuvastatin 20 mg") AND ("percutaneous coronary intervention" OR "PCI")(moderate intensity statins plus ezetimibe OR Atorvastatin plus Ezetimibe OR Rosuvastatin plus Ezetimibe OR Simvastatin plus Ezetimibe OR Pravastatin plus Ezetimibe OR Lovastatin plus Ezetimibe OR Fluvastatin plus Ezetimibe) AND (high intensity statin OR Atorvastatin OR Rosuvastatin) AND (percutaneous coronary intervention)  (moderate intensity statins plus ezetimibe OR Atorvastatin plus Ezetimibe OR Rosuvastatin plus Ezetimibe OR Simvastatin plus Ezetimibe OR Pravastatin plus Ezetimibe OR Lovastatin plus Ezetimibe OR Fluvastatin plus Ezetimibe) AND (high intensity statin OR Atorvastatin OR Rosuvastatin) AND (PCI OR Percutaneous Coronary Intervention OR PCA OR Percutaneous Coronary Angioplasty OR Balloon Angioplasty OR Coronary Angioplasty OR Coronary Stenting OR Primary PCI OR Stent Placement OR Angioplasty with Stenting OR PTCA OR Percutaneous Transluminal Coronary Angioplasty OR Interventional Cardiology Procedure OR Coronary Intervention OR Cardiac Catheterization OR Endovascular Revascularization) | 164 |
| Cochrane | moderate intensity statins plus ezetimibe AND high intensity statins AND percutaneous coronary intervention  statins plus ezetimibe AND percutaneous coronary intervention  (Atorvastatin plus Ezetimibe OR Rosuvastatin plus Ezetimibe OR Simvastatin plus Ezetimibe OR Pravastatin plus Ezetimibe OR Lovastatin plus Ezetimibe OR Fluvastatin plus Ezetimibe) AND (Atorvastatin OR Rosuvastatin) AND percutaneous coronary intervention  (moderate-intensity statin OR atorvastatin OR rosuvastatin OR pravastatin OR fluvastatin OR lovastatin) AND (ezetimibe) AND (high-intensity statin OR atorvastatin 40 mg OR rosuvastatin 20 mg) AND (percutaneous coronary intervention OR PCI)  ("moderate-intensity statin" OR "atorvastatin" OR "rosuvastatin" OR "pravastatin" OR "fluvastatin" OR "lovastatin") AND ("ezetimibe") AND ("high-intensity statin" OR "atorvastatin 40 mg" OR "rosuvastatin 20 mg") AND ("percutaneous coronary intervention" OR "PCI")(moderate intensity statins plus ezetimibe OR Atorvastatin plus Ezetimibe OR Rosuvastatin plus Ezetimibe OR Simvastatin plus Ezetimibe OR Pravastatin plus Ezetimibe OR Lovastatin plus Ezetimibe OR Fluvastatin plus Ezetimibe) AND (high intensity statin OR Atorvastatin OR Rosuvastatin) AND (percutaneous coronary intervention)  (moderate intensity statins plus ezetimibe OR Atorvastatin plus Ezetimibe OR Rosuvastatin plus Ezetimibe OR Simvastatin plus Ezetimibe OR Pravastatin plus Ezetimibe OR Lovastatin plus Ezetimibe OR Fluvastatin plus Ezetimibe) AND (high intensity statin OR Atorvastatin OR Rosuvastatin) AND (PCI OR Percutaneous Coronary Intervention OR PCA OR Percutaneous Coronary Angioplasty OR Balloon Angioplasty OR Coronary Angioplasty OR Coronary Stenting OR Primary PCI OR Stent Placement OR Angioplasty with Stenting OR PTCA OR Percutaneous Transluminal Coronary Angioplasty OR Interventional Cardiology Procedure OR Coronary Intervention OR Cardiac Catheterization OR Endovascular Revascularization) | 101 |

| Supplementary Table 2 Study Design and Intervention Details | | | | | |
| --- | --- | --- | --- | --- | --- |
| S.No. | First author, Year. | Sample size | Intervention/Control and dosage | Primary Outcome Measure(s) | Follow-up period |
| 1. 1. | Seo Young Sohn. 2025 | 6,784 final patients, 4682 (moderate-intensity atorvastatin + ezetimibe), 2102 (high-intensity atorvastatin) | Intervention: moderate-intensity atorvastatin (10 mg and 20 mg) + ezetimibe (10 mg) Control: high-intensity atorvastatin (40 and 80 mg) | Myocardial infarction (MI), Cardiovascular death, coronary revascularization and ischemic stroke | Mean 4.1 years |
| 1. 2. | Seung-Jun Lee. 2024 | 31,966 final patients, 6,164 (Combination lipid-lowering therapy), 25,802 (High-intensity atorvastatin monotherapy) | Intervention: Atorvastatin 20 mg plus ezetimibe 10 mg. Control: Atorvastatin 40-80 mg | Composite of cardiovascular death, myocardial infarction (MI), coronary artery revascularization, hospitalization for heart failure and non-fatal stroke | Mean 3 years |
| 1. 4. | Seung-Jun Lee 2023 | 72,050 final patients,10,794 (Combination lipid-lowering therapy), 61,256 (high-intensity statin monotherapy) after stabilized inverse probability of treatment weighting (IPTW). | Intervention: Rosuvastatin 10 mg + ezetimibe 10 mg Control: Rosuvastatin 20 mg. | Composite event of cardiovascular death, Myocardial Infarction (MI), coronary artery revascularization, hospitalization for Heart failure treatment and nonfatal stroke. | Mean 3 years |
| 1. 5. | Juwon Kim 2021 | 20,070 final patients, 922 (moderate-intensity statin + ezetimibe), 19,148 (High-intensity statins) | Intervention: (atorvastatin 10-20 mg or rosuvastatin 5-10 mg plus ezetimibe) Control: (atorvastatin 40-80 mg or rosuvastatin 20 mg) | Major adverse cardiovascular events (MACE) which include coronary artery revascularization, myocardial infarction, stroke. | Mean 12 months |
| 1. 6. | Kihyun Kim 2021 | 9,908 final patients, 233 Atorvastatin + ezetimibe, 4,041 Atorvastatin, 383 Rosuvastatin + ezetimibe, 5,251 Rosuvastatin | Intervention: Atorvastatin 20 mg + ezetimibe 10 mg, Rosuvastatin 10 mg + ezetimibe 10 mg, Control: Atorvastatin 40 mg, Rosuvastatin 20 mg | Major adverse cardiovascular events (MACE), non-fatal MI undergoing PCI, repeat revascularization and ischemic stroke | Mean 2.5 ± 1.1 years |
| 1. 7. | Mi Seon Ji 2016 | 3,520 final patients, 1249 (simvastatin- ezetimibe), 2271 (High-intensity statins) | Intervention: Simvastatin-ezetimibe Control: High intensity statins (atorvastatin 40-80 mg or rosuvastatin 20-40 mg) | Major adverse cardiovascular events (MACE), recurrent nonfatal myocardial infarction (MI), repeat coronary revascularization. | Mean 12 months |
| 1. 8. | Ji-Yong Jang 2024 | 286,817 total patients undergo screening. 10,723 Moderate statins + Ezetimibe, 10,723 High statins after propensity score matching | Intervention: Moderate statins + ezetimibe Control: High statins | Composite of myocardial infarction (MI), ischemic stroke and all-cause mortality | Mean 2.8 years |
| 1. 9. | Eun Ho Choo 2024 | 79,673 patients were given statin or statin + ezetimibe. 7,161 Moderate intensity statins + ezetimibe, 7,161 high intensity statins after propensity score matching | Intervention: simvastatin 20 or 40 mg, lovastatin 40 mg, pravastatin 40 or 80 mg, fluvastatin 80 mg, atorvastatin 10 or 20 mg, rosuvastatin 5 or 10 mg and pitavastatin 1,2 or 4 mg with ezetimibe. Control: Atorvastatin 40 or 80 mg and rosuvastatin 20 or 40 mg | Major adverse cardiovascular and cerebrovascular events (MACCEs), all-cause death, revascularization (PCI due to MI, PCI due to angina and coronary artery bypass graft) and ischemic stroke. | Mean 2.7 years |
| 1. 10. | Jong-II Park 2023 | 2,497 final patients, 1,258 Moderate intensity statins + ezetimibe, 1,239 high intensity statins monotherapy | Intervention: rosuvastatin 10 mg with ezetimibe 10 mg Control: rosuvastatin 20 mg | Cardiovascular death, major cardiovascular events and nonfatal stroke | Mean 3 years |

**Supplementary Table 3 PICO Criteria Table**

| POPULATION | Patients who underwent percutaneous coronary intervention |
| --- | --- |
| INTERVENTION | Moderate intensity statins plus ezetimibe***** |
| CONTROL | High intensity statins****** |
| OUTCOMES | All cause death, MACE, MI, stroke, and revascularization, Cardiovascular death, diabetes mellitus and rhabdomyolysis. |
| STUDY DESIGN | RCTs, Cohorts |

***Moderate-Intensity Statins: Atorvastatin (10-20 mg), Rosuvastatin (5-10 mg), Simvastatin (20-40 mg), Pravastatin (40-80 mg), Lovastatin (40 mg), Fluvastatin (40 mg twice daily or 80 mg daily), Pitavastatin (2-4 mg).**

****High-Intensity Statins: Atorvastatin (40-80 mg), Rosuvastatin (20-40 mg).**

**Supplementary Figures**

**Supplementary Figure 1: Leave-one-out meta-analysis for MACE outcomes**

**
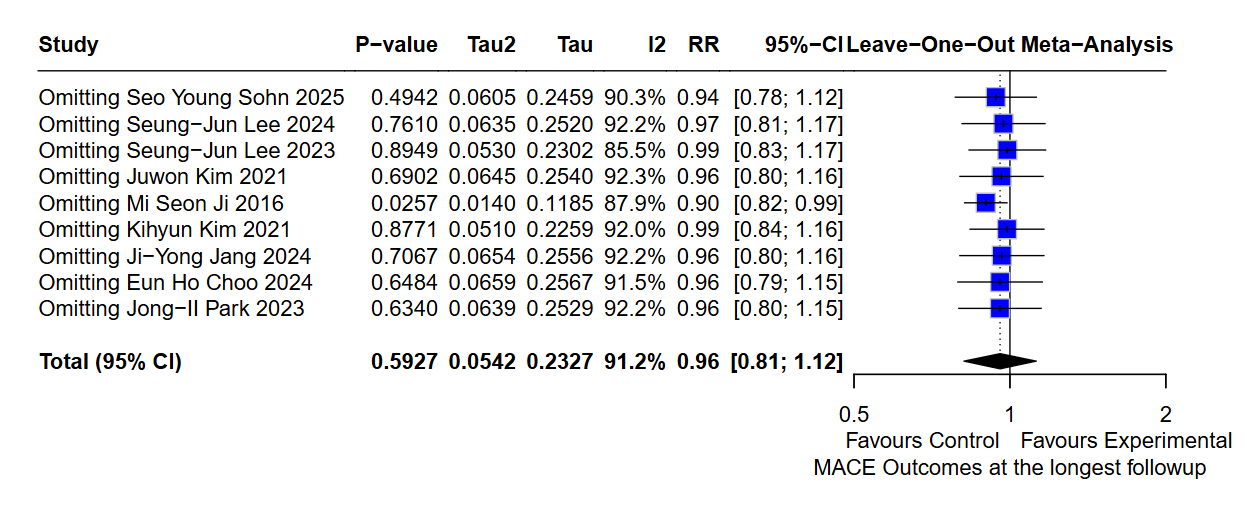
**

**Supplementary Figure 2: Leave-one-out meta-analysis for the ACD outcomes**

**
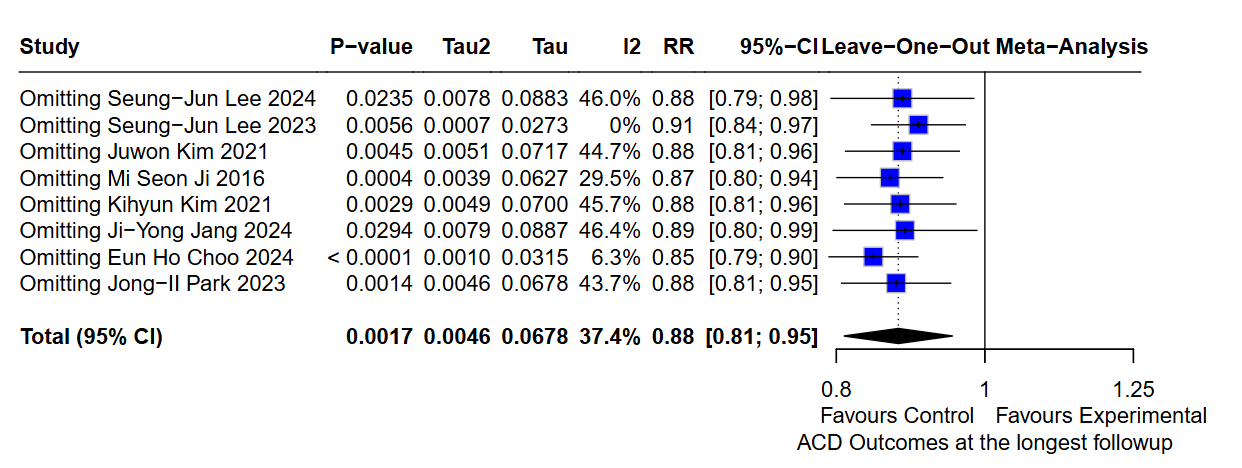
**

**Supplementary Figure 3: Leave-one-out meta-analysis for cardiovascular death**

**
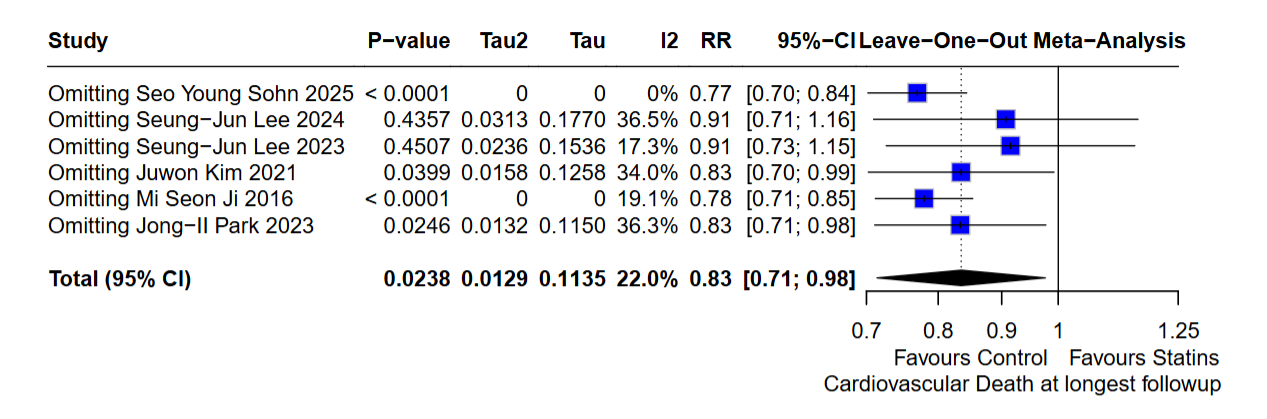
**

**Supplementary Figure 4: Leave-one-out meta-analysis for the stroke**
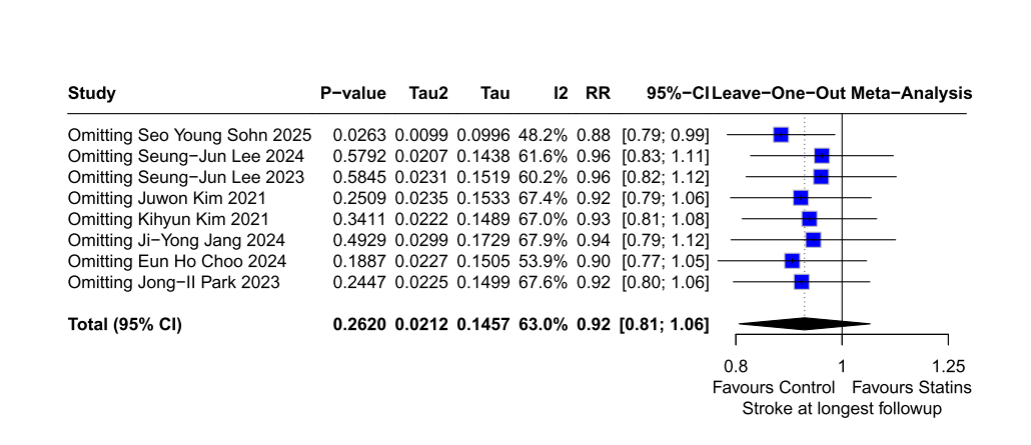


**Supplementary Figure 5: Leave-one-out meta-analysis for MI at longest follow-up**


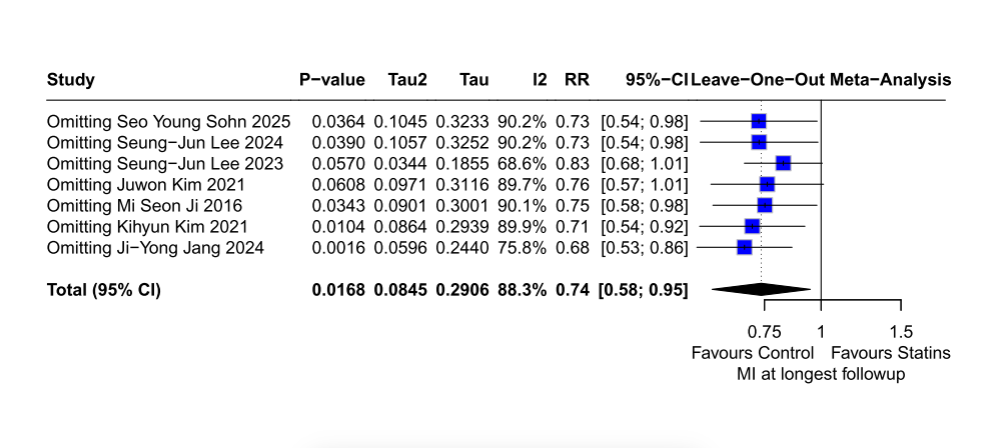


**Supplementary Figure 6: Leave-one-out meta-analysis for revascularization**

**
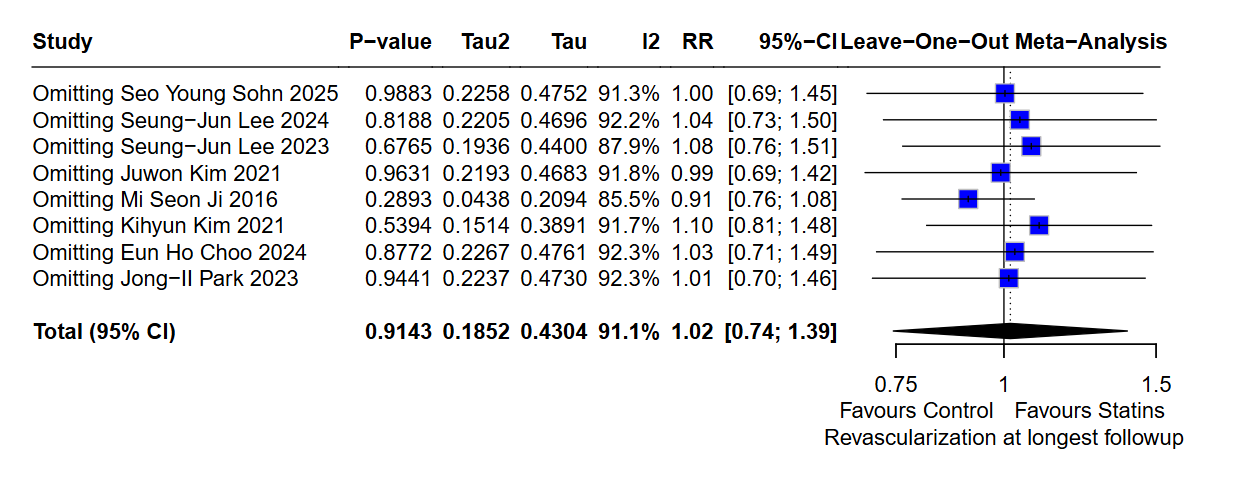
**
